# Genetic and molecular characterization of H9N2 and H5 avian influenza viruses from live poultry markets in Zhejiang Province, eastern China

**DOI:** 10.1038/srep17508

**Published:** 2015-12-02

**Authors:** Haibo Wu, Xiuming Peng, Xiaorong Peng, Linfang Cheng, Xiangyun Lu, Changzhong Jin, Tiansheng Xie, Hangping Yao, Nanping Wu

**Affiliations:** 1 State Key Laboratory for Diagnosis and Treatment of Infectious Diseases, Collaborative Innovation Center for Diagnosis and Treatment of Infectious Diseases, the First Affiliated Hospital, School of Medicine, Zhejiang University, 310003 Hangzhou, China

## Abstract

Live poultry markets (LPMs) are a key source of reassorted avian influenza viruses (AIVs) because of the density of terrestrial and aquatic poultry and the frequency of AIV infection. H9N2 viruses are prevalent in terrestrial poultry throughout Asia and have been isolated from poultry outbreaks worldwide. They infect both avian and mammalian species and may be significant donors of genetic material to emerging human pathogens. LPMs in Zhejiang Province were surveyed from 2013–2014 for AIVs. Three hundred seventy-four (374) AIV strains were isolated from 3,328 samples. Whole–genome sequencing and phylogenetic analyses were performed. We identified a novel H9N2 virus genotype that had undergone reassortment with gene segments from Qa/HK/G1/97–like, Ck/BJ/1/94–like, and Dk/HK/Y439/97–like viruses. Phylogenetic analyses suggested the H9N2 viruses had undergone reassortments with other AIV subtypes. The results also suggested that two different clades (2.3.2 and 2.3.4.6) of H5 viruses were co–circulating in Zhejiang Province. Given that reassorted H5 AIVs were detected in geese and ducks, it is possible that apparently healthy birds contribute to emerging H5 AIVs. Continued surveillance is required in poultry in eastern China.

Many previous studies have demonstrated that H9N2 influenza viruses are established in the terrestrial poultry throughout east Asia^1^,^2^,^3^. The first H9N2 avian influenza outbreak caused by A/Chicken/Beijing/1/94 (Ck/BJ/1/94)–like viruses was reported in China in 1994. Previous studies have shown that terrestrial poultry (especially chickens and quails) play an important role in expanding the host range for avian influenza viruses (AIVs)^1^,^4^,^5^. The H9N2 virus has also played an important role in virus reassortment to generate novel AIVs, including a novel reassortant H7N9 influenza virus associated with human deaths but with no apparent outbreaks in poultry or wild birds^6^. H9N2 AIVs continue to be prevalent in poultry and reassort with other AIV subtypes in mainland China^7^,^8^. In 1999 and 2003, human infections with H9N2 AIVs were reported in Hong Kong and mainland China, respectively^9^,^10^. The circulation of H9N2 influenza viruses throughout China along with their ability to infect mammals and the potential for future reassortment raises concern about their pandemic potential^11^.

Since 2003, the H5N1 highly pathogenic avian influenza (HPAI) viruses have emerged in Asian countries. The HPAI viruses have caused severe epidemics in poultry and resulted in substantial damage to the poultry industry^12^,^13^. As of 17 July 2015, 844 human cases of HPAI infection had been reported to WHO, and of these 449 (53.2%) were fatal^14^. On 6 May 2014, a novel H5N6 AIV was reported in China, and linked to the death of a 49-year-old man, believed to be the world’s first human infected with an H5N6 AIV^15^. The persistent introduction of H5 AIVs into humans and a lack of pre-existing immunity to H5 AIVs suggest that the emergence of a pandemic human influenza virus is possible. Recently, many novel H5 AIVs (H5N6 and H5N8), were first isolated from poultry in eastern China^16^,^18^, and have subsequently spread as HPAI viruses from China to other countries^19^,^20^. However, the most recent prevalence of H5 AIVs in poultry in the Zheijang Province in eastern China has not been described.

Live poultry markets (LPMs) are where domestic poultry (including chickens, ducks, and geese) are slaughtered and sold to households in China and other countries^21^,^22^. LPMs are considered to be a major source of AIV dissemination, sites for potential influenza virus reassortment, and for cross-species transfer of AIVs^8^,^21^,^23^. Previous studies have shown that LPMs in Hong Kong and mainland China were closely linked to the H5N1, H9N2, and H7N9 infections in humans in 1997, 1999, and 2013 respectively^6^,^11^,^24^. Given the critical role of LPMs in viral dissemination, active surveillance, particularly for H9N2 and H5 AIVs, can be an early warning system for AIV outbreaks^25^,^26^. To this end, a survey was conducted in the LPMs of Zheijang Province, eastern China, over a period of 24 months to identify AIVs circulating in poultry. Three hundred and seventy-four (374) AIVs were isolated from apparently healthy poultry, and whole–genome sequencing and phylogenetic analyses were performed. The results suggested reassortment between AIVs from different avian species. The continued circulation of these viruses may pose potential threats for humans.

## Results

### Virus isolation

Swab samples were collected from January 2013 to December 2014 in LPMs throughout Zhejiang Province (eastern China). Three hundred and seventy–four (374) strains of AIVs were isolated. Of these, 212 were isolated from 2031 chicken samples (isolation rate: 10.44%); 101 were isolated from 929 duck samples (10.87%); 25 were isolated from 50 goose samples (50.00%); 25 were isolated from 255 pigeon samples (9.80%); and 11 were isolated from 63 quail samples (17.46%). On average, AIVs were isolated from 11.24% (374/3328) of the swab samples. Given the seasonal nature of influenza outbreaks, we also compared the rate at which AIVs were isolated by month. The percentage of AIV positive swab samples ranged from 1.59–22.92% per month in LPMs in Zheijang Province (Figure S1).

### Hemagglutinin (HA) and neuraminidase (NA) subtypes identified in poultry

We next characterised the HA/NA subtypes of the isolated strains. Within the 374 LPM isolates, 10 HA subtypes (including H1, H2, H3, H4, H5, H6, H7, H9, H10 and H11 subtype), eight NA subtypes (including N1, N2, N3, N4, N6, N7, N8 and N9), and 26 AIVs subtypes overall were identified. The primary subtypes were H3 and H9 which accounted for 29.4% and 31.8% of isolated viruses, respectively. The epidemiologic information for all 374 AIVs is provided in Table 1.

### Phylogenetic analysis of H9N2 viruses

**Figure 1:**
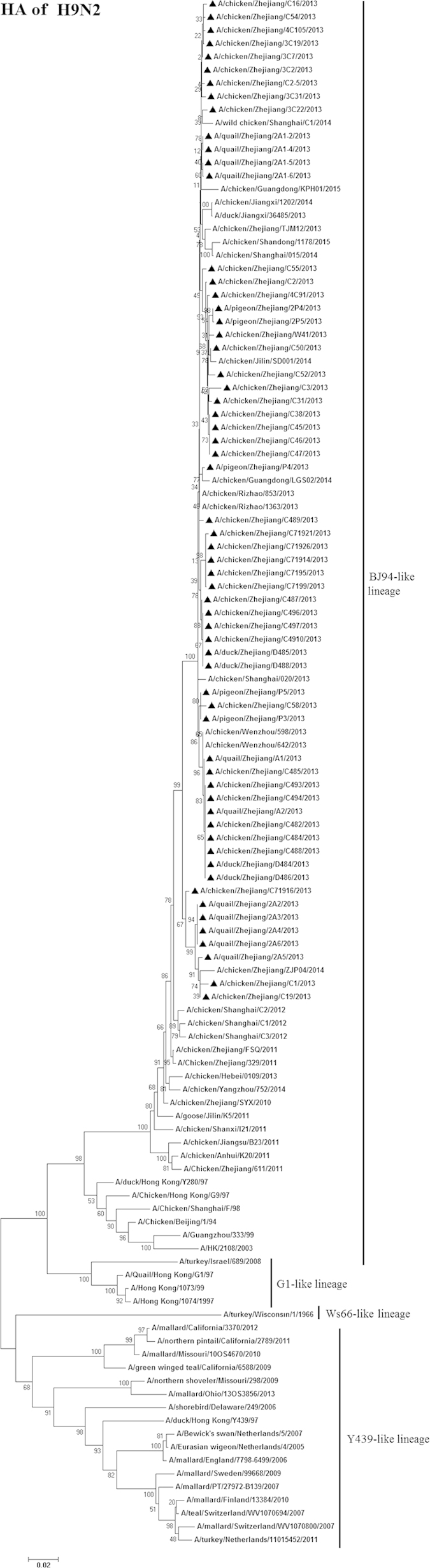
Phylogenetic analysis of the HA segment (positions 20–1660) of H9N2 avian influenza viruses compared to reference influenza viruses. The tree was created by the maximum likelihood method and bootstrapped with 1,000 replicates using the MEGA6 package. Chinese avian influenza viruses from poultry in this study are highlighted by triangles. Scale bar represents the distance unit between sequence pairs.

We next chose 79 isolates that were representative of the 118 H9N2 viruses, based on when and where the strains were isolated, for sequencing and more in depth characterisation. Of the 118 samples positive for H9N2 AIV, two contained multiple HA subtypes (H9N2/H3N2 and H9N2/H7N9), which was confirmed by plaque-purification. All eight gene segments of the 79 selected H9N2 viruses were sequenced. Based on the HA gene, the 79 H9N2 viruses were part of the lineage represented by the reference strains A/duck/Hong Kong/Y280/97 (H9N2) (Dk/HK/Y280/97), Ck/BJ/1/94, and A/Chicken/Shanghai/F/98 (H9N2) (Ck/SH/F/98; Fig. 1 and Table S1). Compared to Dk/HK/Y280/97, the homologies of the HA nucleotide sequences and deduced HA amino acid sequences of the 79 isolates ranged from 90.8–92.0% and 91.7–93.7%, respectively.

**Figure 2:**
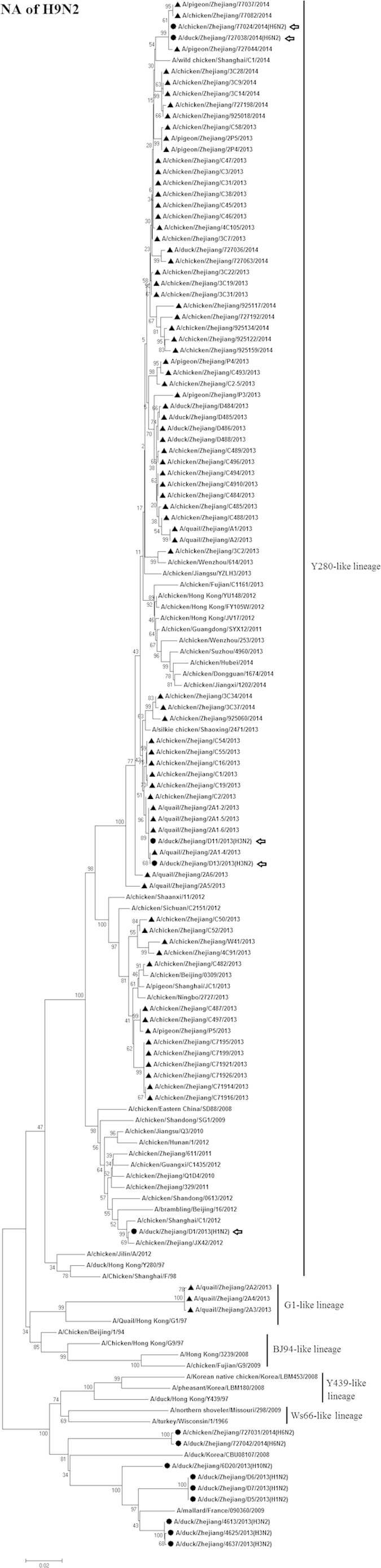
Phylogenetic analysis of the NA segment (positions 25–1375) of H9N2 avian influenza viruses compared to reference influenza viruses. The tree was created by the maximum likelihood method and bootstrapped with 1,000 replicates using the MEGA6 package. Chinese avian influenza viruses from poultry in this study are highlighted by triangles. Scale bar represents the distance unit between sequence pairs. The arrows indicate reassortments between H9N2 and other avian influenza viruses.

Based on the NA gene sequence, there were two different N2 genetic groups circulating simultaneously in poultry in Zhejiang Province. One group contained the A/quail/Zhejiang/2A2/2013, A/quail/Zhejiang/2A3/2013, and A/quail/Zhejiang/2A4/2013 viruses, which formed the lineage represented by A/Quail/Hong Kong/G1/97 (Qa/HK/G1/97; Fig. 2). The other group consisted of the remaining 76 isolated strains, which represent the Dk/HK/Y280/97–like lineage.

Phylogenetic analyses of the remaining six genes polymerase basic protein 2 (PB2), polymerase basic protein 1 (PB1), polymerase acidic protein (PA), nucleocapsid protein (NP), matrix protein (M), and nonstructural protein (NS), indicated that these gene segments had more diversified sources than the HA genes, suggesting that the 79 isolated H9N2 viruses had undergone extensive reassortment. The PB1, NS, and NP genes of A/quail/Zhejiang/2A3/2013, A/quail/Zhejiang/2A4/2013, and A/quail/Zhejiang/2A5/2013 viruses were in the same clade as Qa/HK/G1/97 (Table S1). However, the phylogenetic tree suggested that the PA genes of A/quail/Zhejiang/2A4/2013 and A/quail/Zhejiang/2A5/2013 belonged to the A/Duck/Hong Kong/Y439/97 (H9N2) (Dk/HK/Y439/97) lineage. Phylogenetic analysis of the M and PB2 genes showed that all 79 H9N2 viruses obtained in our study were closely related to the Qa/HK/G1/97 lineage.

In the present study, we assigned H9N2 virus genotypes as previously described^7^,^27^,^28^,^29^,^30^. Two H9N2 influenza virus genotypes were identified. Of these, one was the S genotype (76 isolates, represented by A/chicken/Zhejiang/C1/2013), which contained the O genotype, except for PB2 as proposed by Gu *et al.*^31^. The second genotype identified in this study was a novel genotype, which has likely undergone reassortment with gene segments from Qa/HK/G1/97–like, Ck/BJ/1/94–like, and Dk/HK/Y439/97–like viruses. This genotype has not been published previously and includes the strains A/quail/Zhejiang/2A2/2013, A/quail/Zhejiang/2A3/2013, and A/quail/Zhejiang/2A4/2013 (Table S1).

### Molecular characterization of H9N2 viruses

Having established the genetic context of the isolated AIVs, we next characterised the strains at a molecular level. It is widely known that the addition of amino acids at HA cleavage sites such as arginine (R) and lysine (K) can turn LPAI into HPAI in the H5 and H7 subtypes^32^. Based on the deduced amino acid sequences of the HA genes, the HA cleavage site pattern of the 79 H9N2 strains is PSR(K)SSR/GL, which has a monobasic cleavage site, indicating low pathogenicity in poultry.

We also analysed the amino acids at receptor–binding sites of HA, specifically 109(Y), 161(W), 163(T), 191(N), 198(T/A/V), 202(L), and 203(Y). Considering the specificity of the receptor–binding sites of the 79 H9N2 strains, we selected sequences from 10 of the 79 strains based on when and where the strains were isolated (Table S2). The amino acids at positions 232–237 were “NGLMGR” in all 79 viruses. The receptor–binding sites L234 and G236 were similar across all the viruses, suggesting that the 79 H9N2 viruses would preferentially bind to the α2–6–linked sialic acid receptors that are predominant in humans^33^. The Q234L mutation (Q226L, H3 numbering system) in the HA protein might also contribute to pathogenicity in mammals, as this substitution increases the affinity of the AIVs for α2–6–linked sialic acid receptors found in the mouse and human respiratory tract^34^.

In HA, seven potential glycosylation sites specifically positions 29, 141, 145, 298, 305, 313, 492, and 551, were identified in the H9N2 viruses, based on the definition of a specific polypeptide for *N*–linked glycosylation being Asn–X–Ser/Thr, where X can be any amino acid except proline (P) or aspartic acid (D)^35^. With the exception of the A/quail/Zhejiang/2A2/2013, A/quail/Zhejiang/2A3/2013, and A/quail/Zhejiang/2A4/2013 viruses, three amino acids (TEI; sites 63–65) were deleted at the NA stalk region, as previously described^7^,^24^,^27^. As we reported previously^8^, the deletion of the “TEI” amino acid sequence at the NA stalk region also occurred in the H1N2 virus. In addition, two amino acids (QN; sites 39–40) were deleted at the NA stalk region of three H9N2 viruses isolated from quails.

The NA inhibitors oseltamivir and zanamivir are effective antiviral drugs for treatment and prophylaxis of influenza infections. The His275Tyr mutation is the molecular marker of oseltamivir resistance, and previous studies predicted that mutant viruses would be less viable than sensitive ones^36^. The His275Tyr mutation was not observed in the NA of these 79 H9N2 AIVs. The Val27Ala and Ser31Asn mutations in the M2 protein, which are associated with amantadine resistance^37^, were observed in the M2 protein of all 79 selected H9N2 AIVs in this study.

### Phylogenetic analysis of other viruses

We also isolated one H1N2, two H3N2, and two H6N2 AIVs. Phylogenetic analysis of the N2 genes from these viruses indicated that these viruses were closely related to the H9N2 strains that had been circulating in Zheijang Province, eastern China (Fig. 2). These findings indicated that reassortment events between H1 and H9N2, H3 and H9N2, and H6 and H9N2 likely occurred in poultry.

### Phylogenetic analysis of H5 viruses

**Figure 3:**
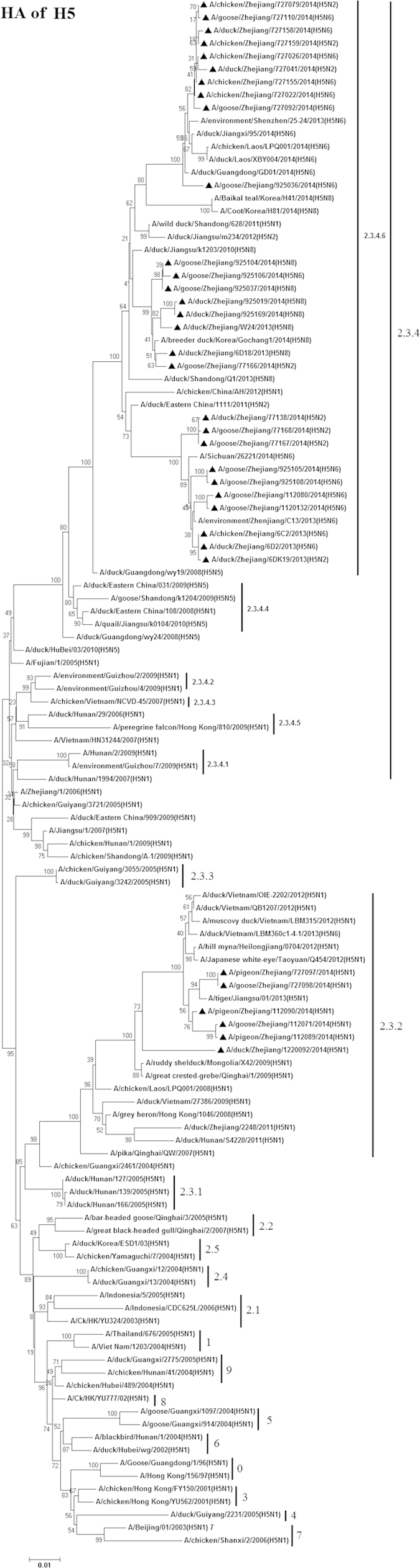
Phylogenetic analysis of the HA segment (positions 1–1644) of the H5 avian influenza viruses compared to reference influenza viruses. The tree was created by the maximum likelihood method and bootstrapped with 1,000 replicates using the MEGA6 package. Chinese avian influenza viruses from poultry in this study are highlighted by triangles. Scale bar represents the distance unit between sequence pairs.

**Figure 4:**
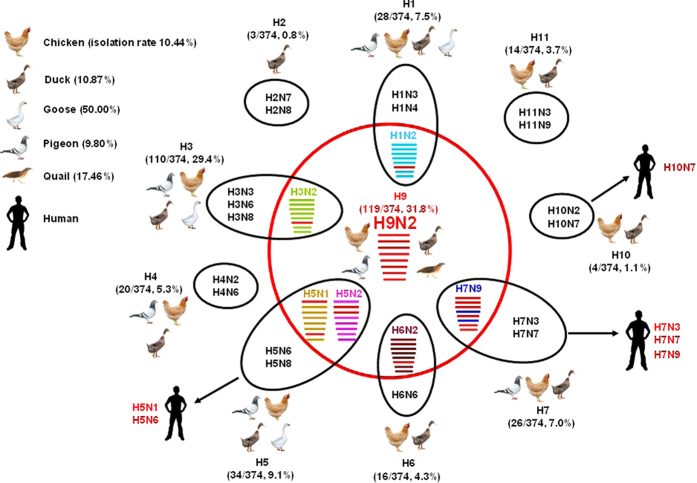
Schematic illustration of the reassortment process of avian influenza viruses in poultry from live poultry markets of Zheijang Province, eastern China. The H9N2 avian influenza viruses donated internal genes to other viruses. The eight gene segments (from top to bottom) in each virus are PB2, PB1, PA, HA, NP, NA, M, and NS. Each color represents a separate virus background. The simplified schematic illustration is based on nucleotide-distance comparison and phylogenetic analysis. The arrows indicate subtypes of avian influenza viruses that can cause human infection.

Whole–genome sequencing and phylogenetic analyses of 34 H5 viruses was performed. Based on the HA genes, there were two different clades (2.3.2 and 2.3.4.6) of viruses circulating simultaneously in Zhejiang Province (Fig. 3 and 4 and Table S3). The group belonging to clade 2.3.2 consisted of 3 strains isolated from pigeons, 1 strain from duck, and 2 strains from geese. The remaining 28 strains belonged to a distinct group, clade 2.3.4.6 (a variant clade 2.3.4) which was proposed separately by Gu *et al.* and Wong *et al.*^19^,^38^ and is closely related to 2014 H5N8 and H5N6 HPAI viruses circulating in South Korea and Laos, respectively^19^,^20^. Previous studies have shown that H5 viruses from clade 2.3.2 have been circulating widely in poultry and wild birds in China since 2007^23^, and the H5 viruses in clade 2.3.4.6 have been the prevalent lineage in chickens since 2009^38^, and these viruses were circulating through chickens (H5 virus isolation rate 0.74%, 5/679), pigeons (6.52%, 3/46), ducks (4.9%, 5/102), and geese (30%, 15/50), in eastern China. Geese had the highest H5 AIVs isolation rate among eastern Chinese poultry.

In this study, we were able to isolate 15 novel reassortant H5 viruses, with four NA subtypes (Table 1). There were five H5N1 isolates from pigeons and geese. Phylogenetic analysis of the N1 genes shows that these viruses were closely related to H5N1 strains that circulated in China and Vietnam at the same time (during 2012–2013). There were eight H5N2 isolates. Phylogenetic analysis of the N2 genes showed that they belonged to three different clades, but were not in the H9N2–like category. There were 14 H5N6 isolates. Phylogenetic analysis suggested that they formed two different genetic groups that circulated simultaneously in Zhejiang Province. The first group consisted of six strains and was most closely related to the novel 2014 H5N6 influenza virus that caused human infection in China (A/Sichuan/26221/2014(H5N6)). The remaining eight H5N6 viruses belonged to a second group and contained an amino acid deletion in the NA stalk region (positions 59–69). Phylogenetic analysis of the H5N8 stains indicated they were closely related to the 2014 H5N8 HPAI viruses circulating in South Korea, in 2014. This suggested that H5N8 viruses had existed in poultry (including ducks and geese) in eastern China and possibly spread to other countries by migratory birds in recent years^17^.

Phylogenetic analysis of the six internal gene segments, PB2, PB1, PA, NP, M, and NS is shown in Figure S2. The PB2 of four H5N1 strains, A/goose/Zhejiang/112071/2014(H5N1), A/pigeon/Zhejiang/112089/2014(H5N1), A/pigeon/Zhejiang/112090/2014(H5N1), and A/duck/Zhejiang/1220092/2014 (H5N1) belong to a cluster containing the H9N2 viruses from China. The PB1 and PA of A/chicken/Zhejiang/727079/2014(H5N2) were closely related to H9N2–like viruses.

### Molecular characterization of H5 viruses

Based on the deduced amino acid sequences of the H5 HA genes, the HA cleavage site pattern, PL(Q)RER(K)RRKR, suggested that the H5 isolates were HPAI viruses. The amino acids of the H5 AIVs at positions 236–241 and 146–150 were “NGQS(R) GR” and “GVSAA”, respectively. The receptor binding sites of the H5 viruses contained Gln226 and Gly228, which suggested they would preferentially bind to avian–like receptors^17^,^23^. The PB2 protein Lys627Glu mutation has been reported to influence the host range and confer increased virulence for H5N1 viruses in animal models^39^, however this mutation was not observed in the PB2 genes from the H5 viruses analysed in this study.

The deletion of five amino acids at position 80–84 in the NS1 proteins of H5N1 AIVs has been observed more frequently in H5N1 AIVs, which might indicate adaptation to avian species^40^,^41^. This deletion was not observed in the A/goose/Zhejiang/925106/2014(H5N6), A/goose/Zhejiang/925037/2014(H5N8), A/goose/Zhejiang/925104/2014(H5N8), A/duck/Zhejiang/925019/2014 (H5N8), A/duck/Zhejiang/925169/2014(H5N8), A/duck/Zhejiang/6D18/2013(H5N8), and A/duck/Zhejiang/W24/2013(H5N8) strains; however it was observed in the remaining 27 H5 viruses. These results indicated that the isolated H5 viruses underwent frequent reassortment with other viruses. All 34 H5 viruses contained the NS1 Pro42Ser mutation, which is associated with increased virulence in mice^42^.

### Evaluating pathogenicity in mice

To evaluate the pathogenicity and replication potential of the isolated viruses in mammalian hosts, we infected mice with one of two H9N2 AIV strains isolated from chickens or quail (A/chicken/Zhejiang/C1/2013 or A/quail/Zhejiang/2A2/2013) or one of four H5 AIV strains isolated from geese (A/goose/Zhejiang/112071/2014 [H5N1], A/goose/Zhejiang/77166/2014 [H5N2], A/goose/Zhejiang/925105/2014 [H5N6], and A/goose/Zhejiang/925037/2014 [H5N8]). All of the viruses were able to replicate without prior adaptation. On day 6 post–inoculation, high titres of virus were detected in the lung, but most of the viruses were not detected in the brain, heart, liver, kidney, and spleen. An isolated virus titre was observed in the liver of mice infected with strain A/goose/Zhejiang/77166/2014 (H5N2) on day 6 post–inoculation. The mice were followed for 14 days post-inoculation to determine the survival rate which ranged from 66.67% (4/6) to 100% (6/6) (Table 2).

**Figure 5:**
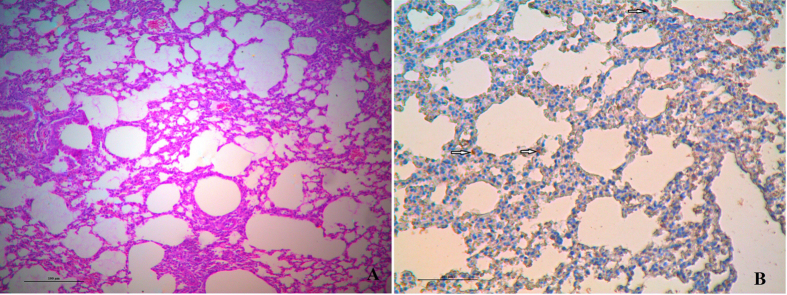
Histology and immunohistochemistry of mice infected with A/goose/Zhejiang/727098/2014(H5N1) at 6 days post–infection. (**A**) Histology of lung sections stained by hematoxylin and eosin from inoculated mice. (**B**) Immunohistochemical detection of virus nucleoprotein in lungs from inoculated mice. Arrows indicate positively stained lung alveolar epithelial cells.

Histopathological analyses showed that 3 days post–inoculation lung tissue had a multifocal mild or moderate interstitial inflammatory hyperaemia and exudative pathological changes. By 6 days post–inoculation, the lesions in the lung tissue became larger, and multiple patchy lesions had fused (Fig. 5A). Immunohistochemistry was also performed to assess whether H5 AIVs infected cells were present in tissues (including bronchial epithelial cells and alveolar epithelial cells) from infected mice 6 days post–inoculation (Fig. 5B). The H5 viruses were able to replicate in both bronchial epithelial cells and alveolar epithelial cells. In addition, the viruses caused severe lung injury in mice, which manifested as congestion and bleeding.

## Discussion

H9N2 viruses are unique among LPAI viruses in that they infect a wide variety of species, including both avian and mammalian species, and they may be significant donors of genetic material to emerging human pathogens^6^. Aquatic birds, including ducks and geese, represent the major natural reservoir of AIVs. In the present study, the isolation rates of H9N2 viruses from chickens (89/2031, 4.38%), pigeons (12/255, 5.33%), and quails (11/63, 17.46%) were higher than those from ducks (6/929, 0.65%) and geese (0/50, 0%), indicating that the H9N2 virus infected terrestrial poultry with greater frequency than aquatic birds. H9N2 viruses may be able to adapt to the human host in terrestrial poultry. Quails and chickens have the molecular characteristics of a potential intermediate hosts for AIVs transmission to humans and could generate new influenza viruses with pandemic potential.

H9N2 viruses have undergone extensive reassortment to create multiple novel genotypes from different lineages in poultry. Previous studies have shown that the Asian H9N2 viruses can be divided into three sublineages represented by Ck/BJ/1/94, Qa/HK/G1/97, and Dk/HK/Y439/97^1^,^43^. Since 1998, the H9N2 genotype H virus has become predominant in chickens. Zhang *et al.* demonstrated that the Ck/BJ/1/94–like H9N2 viruses circulating in poultry in eastern China before 1998 have been gradually replaced by Ck/SH/F/98–like H9N2 viruses, whose genotypes differ from those of viruses isolated in southern China^7^. These results suggested that the H9N2 viruses had undergone multiple reassortments in poultry.

In the past decade, although the vaccination strategy for H5N1 influenza has effectively reduced the mortality caused by HPAI H5 virus infection in poultry in China, the H5 AIVs, including H5N1, H5N2, H5N5, H5N6, and H5N8, will continue to persist for much longer in poultry and remain a potential threat to poultry and human health^23^,^44^,^45^. In the present study, the isolation rates of H5 viruses from chickens (0.30%) and pigeons (1.18%) were lower than those from ducks (10.10%) and geese (30%), which indicated that the H5 viruses infected aquatic birds with greater frequency than terrestrial poultry. Geese had the highest H5 AIVs isolation rate among the different eastern Chinese poultry. Our previous results showed that reassorted H5 isolates were highly pathogenic in chickens and moderately pathogenic in mice^16^,^17^,^46^. Given that the reassorted H5 AIVs were detected in geese and domestic ducks in LPMs, it is possible that apparently healthy geese and domestic ducks contribute to creating H5 AIVs.

In summary, we isolated 374 AIV strains from poultry in LPMs in Zhejiang Province, eastern China. These strains displayed complexity and diversity of molecular characteristics. Their phylogenetic and genotype analysis suggested that H9N2 viruses undergo extensive reassortments to generate multiple novel genotypes from different lineages in poultry. We identified a novel H9N2 virus genotype that had undergone reassortment with gene segments from Qa/HK/G1/97–like, Ck/BJ/1/94–like, and Dk/HK/Y439/97–like viruses. In addition, reassorted H5 AIVs were detected in geese and domestic ducks. The results also suggested that two different clades (2.3.2 and 2.3.4.6) of H5 viruses were co–circulating in Zhejiang Province. As LPMs play an important role in the dissemination of AIVs, active surveillance to monitor HPAI and LPAI viruses in LPMs should be carried out as an early warning system for AIV outbreaks. The widespread distribution of the LPAI virus H9N2 in poultry and its potential for reassortment are a credible threat for human infections in the future.

## Methods

### Virus isolation

Between January 2013 and December 2014, 3328 cloacal swabs were collected from apparently healthy poultry, including chickens (n = 2031), ducks (n = 929), pigeons (n = 255), quails (n = 63), and geese (n = 50) in 25 LPMs in Hangzhou, Shaoxing, Jiaxing, and Anji, Zhejiang Province, eastern China. Each swab was eluted with 2.0 mL phosphate–buffered saline (PBS) containing 0.2% bovine serum albumin, 4 × 10^6^ U/L penicillin G, and 400 mg/L streptomycin sulfate. A 0.22–μm filter was used to sterilize the samples, which was then inoculated into the allantoic cavities of 9–day–old specific pathogen–free embryonated eggs as described previously^8^. After incubation at 37 °C for 72 h, the allantoic fluid was harvested and tested using the HA assay. Aliquots of the allantoic fluid containing virus were stored at –80 °C for further analysis. The subtypes of the virus isolates were determined by conventional HA inhibition (HI) and NA inhibition (NI) assays, according to the Office International des Epizootics (OIE) manual^47^. Samples thought to contain mixed infections were confirmed by plaque-purification. All of the experiments with the AIVs were performed in a Biosafety Level 3 laboratory.

### RNA extraction and PCR amplification

RNA was extracted from the HA–positive allantoic fluid samples using a viral RNA mini kit (Qiagen, Germany) per the manufacturer’s instructions. Reverse transcription was performed using the Uni12 primer: 5ʹ–AGCAAAAGCAGG–3′. Reverse transcriptase–polymerase chain reaction (RT–PCR) was conducted using a One–Step RNA PCR Kit (TaKaRa, China). All segments were amplified with segment–specific primers described previously^48^. The PCR products were purified using the Agarose Gel DNA Fragment Recovery Kit Ver. 2.0 (TaKaRa, China).

### Sequencing and phylogenetic analysis

The fragments were sequenced using a Big Dye Terminator V.3.0 Cycle Sequencing Ready Reaction kit (ABI, Foster City, CA), per the manufacturer’s instructions. All eight gene segments of the AIV field isolates were sequenced and compared with the classical reference viruses. The classical reference viruses were selected based from the literature^7^,^26^,^27^,^49^; for H9N2 viruses, we selected Ck/BJ/1/94, Ck/SH/F/98, Qa/HK/G1/97, Y439 = Dk/HK/Y439/97, DK/HK/d73/76, and Ck/Kor/323/96 as the reference sequences. The sequences were analysed using BioEdit version 7.0.9.0 DNA software. Phylogenetic trees were constructed using molecular evolutionary genetics analysis (MEGA) software version 6, applying the maximum likelihood method and Tamura–Nei model with bootstrap analysis (1,000 replicates)^50^. The reference sequences of the strains used in this study were obtained from the Influenza Sequences Database (http://www.ncbi.nlm.nih.gov). The nucleotide sequences were deposited into GenBank under the accession numbers: KP063194-201, KP412438-61, KF357794-821, KJ439814-901, KF357746-77, KU042087-886, KF357822-57, KT423122-77, and KT589138-321.

### Animal study

To evaluate virus pathogenicity and replication potential in mammalian hosts, 15 6–week–old female BALB/c mice were intranasally inoculated with 10^6.0^ EID50 of virus. Three (3) mice were sacrificed at 3, 6, and 9 days post–inoculation. Their lungs, brains, hearts, and livers were collected to determine viral distribution in the tissue based on virus titration in embryonated chicken eggs. The survival rate was observed in the remaining six mice over 14 days following inoculation as described previously^23^. The animal studies were performed in accordance with the recommendations of the Office International des Epizooties (OIE)^51^ and approved by the First Affiliated Hospital, School of Medicine, Zhejiang University (No. 2015-015).

### Histology and immunohistology of mouse lung sections

Lung tissues from virus–inoculated mice were fixed in 10% neutral buffered formalin for at least 24 h before processing. The tissues were embedded in paraffin by standard tissue processing procedures. Sections 4 μm thick were cut and fixed on glass slides. Standard hematoxylin and eosin (H&E) staining was carried out. Immunohistochemical staining to detect nucleoprotein antigens in the lungs was performed using a monoclonal antibody against the influenza A virus nucleoprotein (1:100 dilution) at 4 °C overnight. The sections were washed 3 times with PBS and then incubated with HRP–conjugated goat anti–mouse secondary antibody (1:3000 dilutions, Sigma). The sections were developed with 3–3′ diaminobenzidine (DAB) and examined with a light microscope.

## Additional Information

**How to cite this article**: Wu, H. *et al.* Genetic and molecular characterization of H9N2 and H5 avian influenza viruses from live poultry markets in Zhejiang Province, eastern China. *Sci. Rep.* **5**, 17508; doi: 10.1038/srep17508 (2015).

## Supplementary Material

Supplementary Information

## Figures and Tables

**Table 1:** General overview of the avian influenza viruses collected from poultry in live poultry markets in Zhejiang province from 2013–2014.

HA subtypes(Percentage)	AIVsubtypes	The number oftotal strains	The number ofviruses sequenced	Species (The number of strains)
H1(28/374, 7.5%)	H1N2	4	4	Duck(4)
	H1N3	20	4	Chicken(1), Duck(11), Goose(7), Pigeon(1)
	H1N4	4	4	Duck(4)
H2(3/374, 0.8%)	H2N7	2	2	Duck(2)
	H2N8	1	1	Duck(1)
H3(110/374, 29.4%)	H3N2	85	25	Chicken(57), Duck(20), Goose(3), Pigeon(5)
	H3N6	23	6	Chicken(23)
	H3N8	2	2	Duck(2)
H4(20/374, 5.3%)	H4N2	16	16	Chicken(1), Duck(13), Pigeon(2)
	H4N6	4	4	Duck(4)
H5(34/374, 9.1%)	H5N1	6	6	Duck(1), Goose(2), Pigeon(3)
	H5N2	8	8	Chicken(2), Duck(3), Goose(3)
	H5N6	14	14	Chicken(4), Duck(2), Goose(8)
	H5N8	6	6	Duck(4), Goose(2)
H6(16/374, 4.3%)	H6N2	7	7	Chicken(4), Duck(3)
	H6N6	9	9	Chicken(9)
H7(26/374, 7.0%)	H7N3	10	6	Duck(10)
	H7N7	1	1	Chicken (1)
	H7N9	15	10	Chicken(13), Pigeon(2)
H9(119/374, 31.8%)	H9N2	118	79	Chicken(89), Duck(6), Pigeon(12), Quail(11)
	H9N9	1	1	Chicken(1)
H10(4/374, 1.1%)	H10N2	1	1	Duck(1)
	H10N7	3	3	Chicken(3)
H11(14/374, 3.7%)	H11N2	3	3	Duck(3)
	H11N3	6	4	Chicken(3), Duck(3)
	H11N9	5	5	Duck(5)
Total	26	374	231	Chicken(212/374*, 56.7%)
	subtypes	strains	strains	Duck(101/374, 27.0%)
				Goose(25/374, 6.7%)
				Pigeon(25/374, 6.7%)
				Quail(11/374, 2.9%)

Note: Viruses were harvested during surveillance of live poultry markets in Zhejiang, China from January 2013 through December 2014.

**Table 2:** Survival rate and tissue distribution of novel H9N2 and H5 avian influenza viruses in mice.

Virus	Survival rate of mice	Virus replication in experimentally infected mice Virus titers in organs of mice (log_10_ EID_50/_ml)			
		Tissue	3 day	6 day	9 day
A/chicken/Zhejiang/C1/2013(H9N2)	100% (6/6)	Lung	2.5 ± 0.71	3.5 ± 0.71	2.0 ± 0
		Brain			–
		Heart	–	–	–
		Liver	–	–	–
A/quail/Zhejiang/2A2/2013(H9N2)	100% (6/6)	Lung	2.0 ± 0.0	3.5 ± 0.71	2.5 ± 0.71
		Brain	–		–
		Heart	–	–	–
		Liver	–	–	–
A/goose/Zhejiang/112071/2014(H5N1)	83.33% (5/6)	Lung	3.5 ± 0.71	4.5 ± 0.71	4.0 ± 0
		Brain			–
		Heart	–	–	–
		Liver	–	–	–
A/goose/Zhejiang/77166/2014(H5N2)	66.67% (4/6)	Lung	4.0 ± 0	5.0 ± 0	4.5 ± 0.71
		Brain	–	–	–
		Heart	–	–	–
		Liver	–	3.5 ± 0.71	–
A/goose/Zhejiang/925105/2014(H5N6)	100% (6/6)	Lung	3.5 ± 0.71	4.5 ± 0.71	3.0 ± 0
		Brain			–
		Heart	–	–	–
		Liver	–	–	–
A/goose/Zhejiang/925037/2014(H5N8)	83.33% (5/6)	Lung	3.0 ± 0	5.0 ± 0	3.5 ± 0.71
		Brain	–	–	–
		Heart	–	–	–
		Liver	–	–	–

**Notes:** Fifteen (15) 6-week-old female BALB/c mice were intranasally inoculated with 10^6.0^ EID50 of virus. Three (3) mice each were sacrificed on days 3, 6, and 9 post-inoculation. The lungs, brain, heart, and liver were collected for virus titration in embryonated chicken eggs. The survival rate was determined in the remaining six mice 14 days following inoculation. The EID_50_ was determined in embryonated chicken eggs using the method published by Reed and Muench. Values represent mean ± SD.
