# Supplementary material for: Genetic and molecular characterization of H9N2 and H5 avian influenza viruses from live poultry markets in Zhejiang Province, eastern China
Source: Sci Rep. 2015 Dec 2;5:17508. doi: 10.1038/srep17508 (PMC4667249; doi:10.1038/srep17508)
Supplement: Supplementary Information [file srep17508-s1.pdf]

Genetic and molecular characterization of H9N2 and H5 avian influenza viruses from live poultry markets in Zhejiang Province, eastern China

Haibo Wu[1], Xiuming Peng[1], Xiaorong Peng[1], Linfang Cheng[1], Xiangyun Lu[1], Changzhong Jin[1], Tiansheng Xie[1], Hangping Yao[1], and Nanping Wu[1]

1. State Key Laboratory for Diagnosis and Treatment of Infectious Diseases, Collaborative Innovation Center for Diagnosis and Treatment of Infectious Diseases, the First Affiliated Hospital, School of Medicine, Zhejiang University, 310003 Hangzhou, China

Nanping Wu, Corresponding author

State Key Laboratory for Diagnosis and Treatment of Infectious Diseases, Collaborative Innovation Center for Diagnosis and Treatment of Infectious Diseases, the First Affiliated Hospital, School of Medicine, Zhejiang University, 79 Qingchun Road, Hangzhou, Zhejiang, 310003, China.

Tel: +86 571 8723 6580. Email: flwnp2013@163.com.

Supplementary Material:

Figure legends:

Figure S1. Percentage of avian influenza virus positive poultry swabs by month. Cloacal swabs samples were collected from live poultry markets in Zheijang Province, eastern China from 2013–2014. The percentages of the swabs that tested positive for avian influenza virus each month during the collection period are shown.

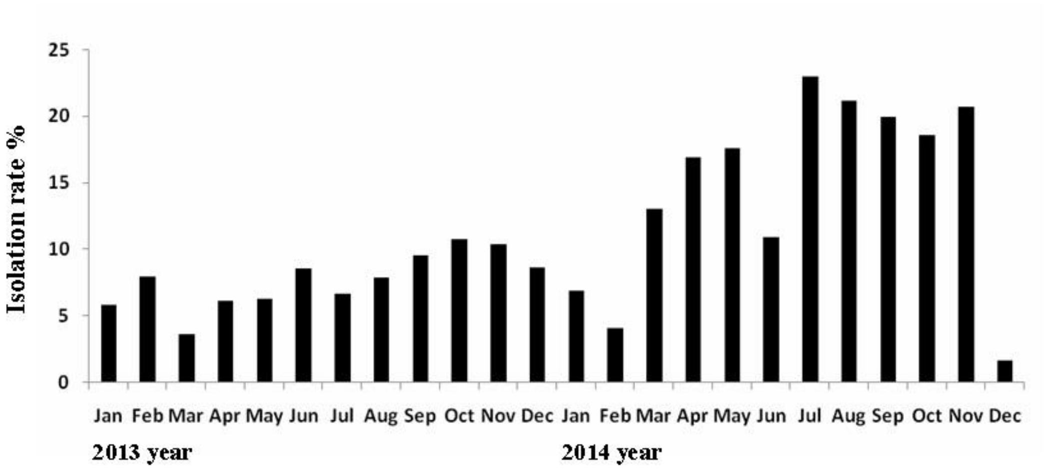
Isolation rate %
25
20
15
10
5
0
Jan Feb Mar Apr May Jun Jul Aug Sep Oct Nov Dec Jan Feb Mar Apr May Jun Jul Aug Sep Oct Nov Dec
2013 year
2014 year

Figure S2. Phylogenetic analysis of the NA (N1, positions 1–1410; N2, 40–1340; N6, 40–1352; N8, 44–1352), PB2 (positions 1–2280), PB1 (positions 16–2268), PA (positions 1–2151), NP (positions: 1–1436), M (positions 29–923), and NS (positions 28–768) segments of the H5 avian influenza viruses compared to reference influenza viruses. The tree was created by the maximum likelihood method and bootstrapped with 1,000 replicates using the MEGA6 package. The H5 viruses from poultry in China identified in this study are highlighted by triangles. The non-H5 viruses from poultry in China identified in this study are highlighted by dots. Scale bar represents the distance unit between sequence pairs.

N1 of H5

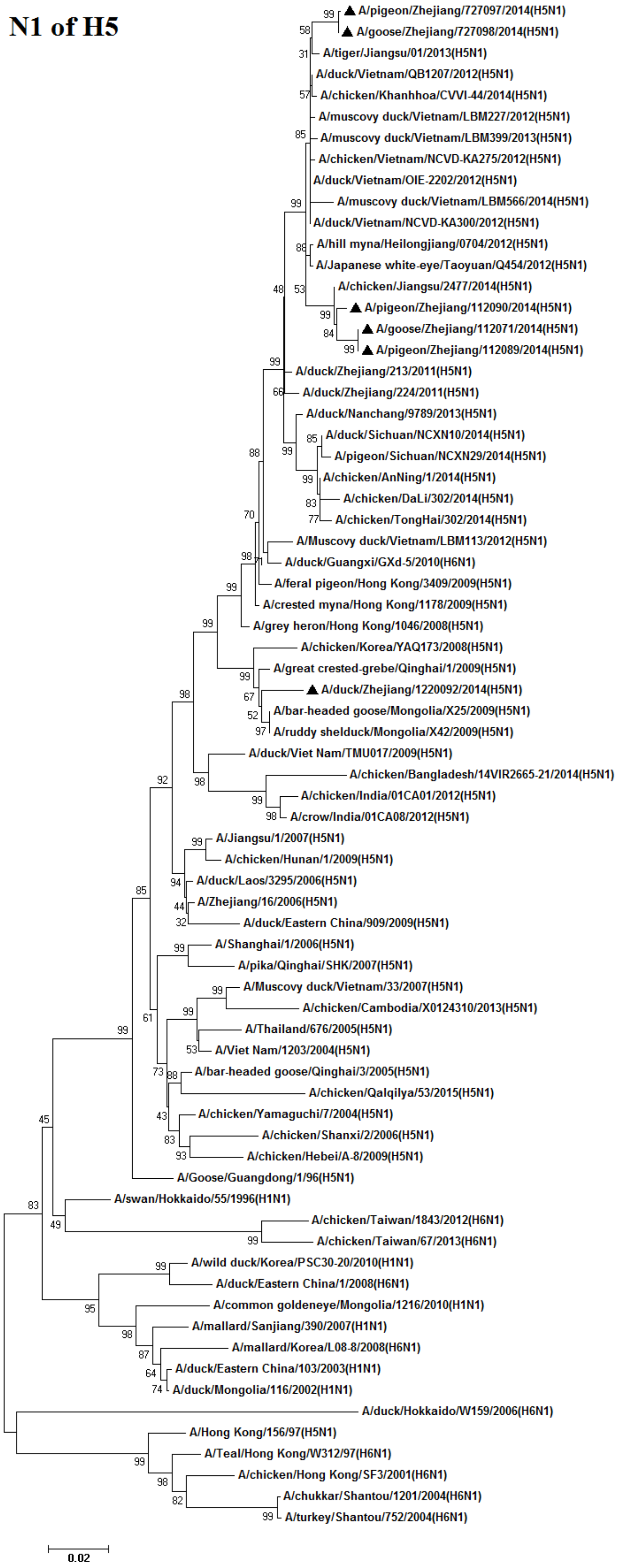
A/pigeon/Zhejiang/727097/2014(H5N1)
A/goose/Zhejiang/727098/2014(H5N1)
A/tiger/Jiangsu/01/2013(H5N1)
A/duck/Vietnam/QB1207/2012(H5N1)
A/chicken/Khanhhoa/CVVI-44/2014(H5N1)
A/muscovy duck/Vietnam/LBM227/2012(H5N1)
A/muscovy duck/Vietnam/LBM399/2013(H5N1)
A/chicken/Vietnam/NCVD-KA275/2012(H5N1)
A/duck/Vietnam/OIE-2202/2012(H5N1)
A/muscovy duck/Vietnam/LBM566/2014(H5N1)
A/duck/Vietnam/NCVD-KA300/2012(H5N1)
A/hill myna/Heilongjiang/0704/2012(H5N1)
A/Japanese white-eye/Taoyuan/Q454/2012(H5N1)
A/chicken/Jiangsu/2477/2014(H5N1)
A/pigeon/Zhejiang/112090/2014(H5N1)
A/goose/Zhejiang/112071/2014(H5N1)
A/pigeon/Zhejiang/112089/2014(H5N1)
A/duck/Zhejiang/213/2011(H5N1)
A/duck/Zhejiang/224/2011(H5N1)
A/duck/Nanchang/9789/2013(H5N1)
A/duck/Sichuan/NCXN10/2014(H5N1)
A/pigeon/Sichuan/NCXN29/2014(H5N1)
A/chicken/AnNing/1/2014(H5N1)
A/chicken/DaLi/302/2014(H5N1)
A/chicken/TongHai/302/2014(H5N1)
A/Muscovy duck/Vietnam/LBM113/2012(H5N1)
A/duck/Guangxi/GXd-5/2010(H6N1)
A/feral pigeon/Hong Kong/3409/2009(H5N1)
A/crested myna/Hong Kong/1178/2009(H5N1)
A/grey heron/Hong Kong/1046/2008(H5N1)
A/chicken/Korea/YAQ173/2008(H5N1)
A/great crested-grebe/Qinghai/1/2009(H5N1)
A/duck/Zhejiang/1220092/2014(H5N1)
A/bar-headed goose/Mongolia/X25/2009(H5N1)
A/ruddy shelduck/Mongolia/X42/2009(H5N1)
A/duck/Viet Nam/TMU017/2009(H5N1)
A/chicken/Bangladesh/14VIR2665-21/2014(H5N1)
A/chicken/India/01CA01/2012(H5N1)
A/crow/India/01CA08/2012(H5N1)
A/Jiangsu/1/2007(H5N1)
A/chicken/Hunan/1/2009(H5N1)
A/duck/Laos/3295/2006(H5N1)
A/Zhejiang/16/2006(H5N1)
A/duck/Eastern China/909/2009(H5N1)
A/Shanghai/1/2006(H5N1)
A/pika/Qinghai/SHK/2007(H5N1)
A/Muscovy duck/Vietnam/33/2007(H5N1)
A/chicken/Cambodia/X0124310/2013(H5N1)
A/Thailand/676/2005(H5N1)
A/Viet Nam/1203/2004(H5N1)
A/bar-headed goose/Qinghai/3/2005(H5N1)
A/chicken/Qalqilya/53/2015(H5N1)
A/chicken/Yamaguchi/7/2004(H5N1)
A/chicken/Shanxi/2/2006(H5N1)
A/chicken/Hebei/A-8/2009(H5N1)
A/Goose/Guangdong/1/96(H5N1)
A/swan/Hokkaido/55/1996(H1N1)
A/chicken/Taiwan/1843/2012(H6N1)
A/chicken/Taiwan/67/2013(H6N1)
A/wild duck/Korea/PSC30-20/2010(H1N1)
A/duck/Eastern China/1/2008(H6N1)
A/common goldeneye/Mongolia/1216/2010(H1N1)
A/mallard/Sanjiang/390/2007(H1N1)
A/mallard/Korea/L08-8/2008(H6N1)
A/duck/Eastern China/103/2003(H1N1)
A/duck/Mongolia/116/2002(H1N1)
A/duck/Hokkaido/W159/2006(H6N1)
A/Hong Kong/156/97(H5N1)
A/Teal/Hong Kong/W312/97(H6N1)
A/chicken/Hong Kong/SF3/2001(H6N1)
A/chukkar/Shantou/1201/2004(H6N1)
A/turkey/Shantou/752/2004(H6N1)
0.02

N2 of H5

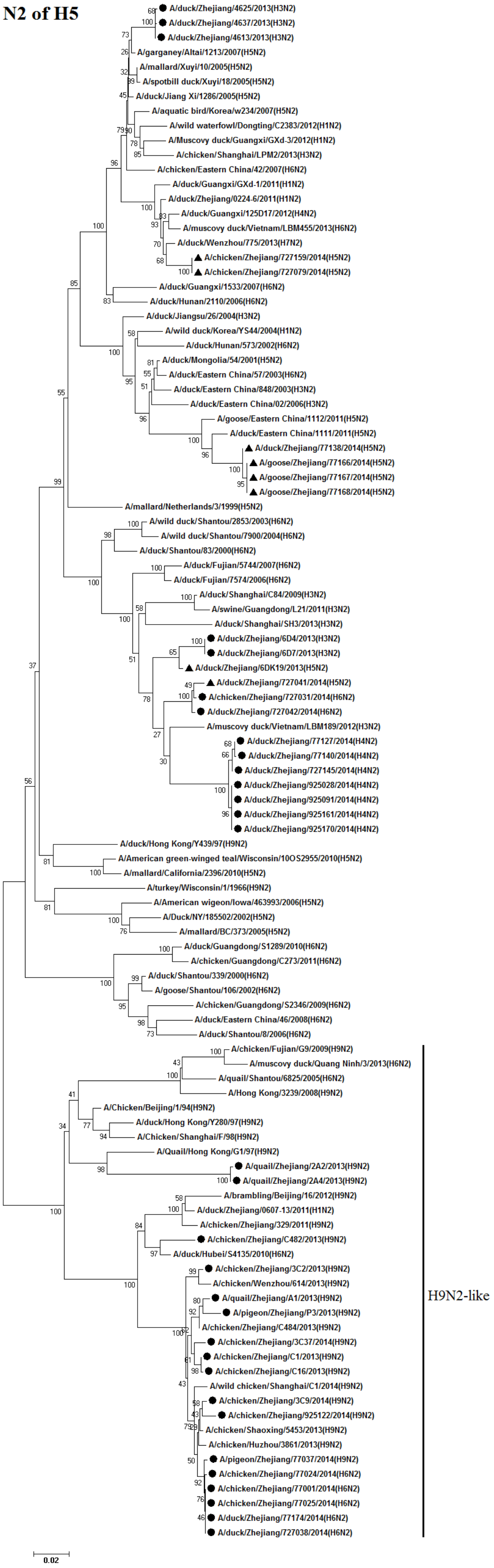
A/duck/Zhejiang/4625/2013(H3N2)
A/duck/Zhejiang/4637/2013(H3N2)
A/duck/Zhejiang/4613/2013(H3N2)
A/garganey/Altai/1213/2007(H5N2)
A/mallard/Xuyi/10/2005(H5N2)
A/spotbill duck/Xuyi/18/2005(H5N2)
A/duck/Jiang Xi/1286/2005(H5N2)
A/aquatic bird/Korea/w234/2007(H5N2)
A/wild waterfowl/Dongting/C2383/2012(H1N2)
A/Muscovy duck/Guangxi/GXd-3/2012(H1N2)
A/chicken/Shanghai/LPM2/2013(H3N2)
A/chicken/Eastern China/42/2007(H6N2)
A/duck/Guangxi/GXd-1/2011(H1N2)
A/duck/Zhejiang/0224-6/2011(H1N2)
A/duck/Guangxi/125D17/2012(H4N2)
A/muscovy duck/Vietnam/LBM455/2013(H6N2)
A/duck/Wenzhou/775/2013(H7N2)
A/chicken/Zhejiang/727159/2014(H5N2)
A/chicken/Zhejiang/727079/2014(H5N2)
A/duck/Guangxi/1533/2007(H6N2)
A/duck/Hunan/2110/2006(H6N2)
A/duck/Jiangsu/26/2004(H3N2)
A/wild duck/Korea/YS44/2004(H1N2)
A/duck/Hunan/573/2002(H6N2)
A/duck/Mongolia/54/2001(H5N2)
A/duck/Eastern China/57/2003(H6N2)
A/duck/Eastern China/848/2003(H3N2)
A/duck/Eastern China/02/2006(H3N2)
A/goose/Eastern China/1112/2011(H5N2)
A/duck/Eastern China/1111/2011(H5N2)
A/duck/Zhejiang/77138/2014(H5N2)
A/goose/Zhejiang/77166/2014(H5N2)
A/goose/Zhejiang/77167/2014(H5N2)
A/goose/Zhejiang/77168/2014(H5N2)
A/mallard/Netherlands/3/1999(H5N2)
A/wild duck/Shantou/2853/2003(H6N2)
A/wild duck/Shantou/7900/2004(H6N2)
A/duck/Shantou/83/2000(H6N2)
A/duck/Fujian/5744/2007(H6N2)
A/duck/Fujian/7574/2006(H6N2)
A/duck/Shanghai/C84/2009(H3N2)
A/swine/Guangdong/L21/2011(H3N2)
A/duck/Shanghai/SH3/2013(H3N2)
A/duck/Zhejiang/6D4/2013(H3N2)
A/duck/Zhejiang/6D7/2013(H3N2)
A/duck/Zhejiang/6DK19/2013(H5N2)
A/duck/Zhejiang/727041/2014(H5N2)
A/chicken/Zhejiang/727031/2014(H6N2)
A/duck/Zhejiang/727042/2014(H6N2)
A/muscovy duck/Vietnam/LBM189/2012(H3N2)
A/duck/Zhejiang/77127/2014(H4N2)
A/duck/Zhejiang/77140/2014(H4N2)
A/duck/Zhejiang/727145/2014(H4N2)
A/duck/Zhejiang/925028/2014(H4N2)
A/duck/Zhejiang/925091/2014(H4N2)
A/duck/Zhejiang/925161/2014(H4N2)
A/duck/Zhejiang/925170/2014(H4N2)
A/duck/Hong Kong/Y439/97(H9N2)
A/American green-winged teal/Wisconsin/10OS2955/2010(H5N2)
A/mallard/California/2396/2010(H5N2)
A/turkey/Wisconsin/1/1966(H9N2)
A/American wigeon/Iowa/463993/2006(H5N2)
A/Duck/NY/185502/2002(H5N2)
A/mallard/BC/373/2005(H5N2)
A/duck/Guangdong/S1289/2010(H6N2)
A/chicken/Guangdong/C273/2011(H6N2)
A/duck/Shantou/339/2000(H6N2)
A/goose/Shantou/106/2002(H6N2)
A/chicken/Guangdong/S2346/2009(H6N2)
A/duck/Eastern China/46/2008(H6N2)
A/duck/Shantou/8/2006(H6N2)
A/chicken/Fujian/G9/2009(H9N2)
A/muscovy duck/Quang Ninh/3/2013(H6N2)
A/quail/Shantou/6825/2005(H6N2)
A/Hong Kong/3239/2008(H9N2)
A/Chicken/Beijing/1/94(H9N2)
A/duck/Hong Kong/Y280/97(H9N2)
A/Chicken/Shanghai/F/98(H9N2)
A/Quail/Hong Kong/G1/97(H9N2)
A/quail/Zhejiang/2A2/2013(H9N2)
A/quail/Zhejiang/2A4/2013(H9N2)
A/brambling/Beijing/16/2012(H9N2)
A/duck/Zhejiang/0607-13/2011(H1N2)
A/chicken/Zhejiang/329/2011(H9N2)
A/chicken/Zhejiang/C482/2013(H9N2)
A/duck/Hubei/S4135/2010(H6N2)
A/chicken/Zhejiang/3C2/2013(H9N2)
A/chicken/Wenzhou/614/2013(H9N2)
A/quail/Zhejiang/A1/2013(H9N2)
A/pigeon/Zhejiang/P3/2013(H9N2)
A/chicken/Zhejiang/C484/2013(H9N2)
A/chicken/Zhejiang/3C37/2014(H9N2)
A/chicken/Zhejiang/C1/2013(H9N2)
A/chicken/Zhejiang/C16/2013(H9N2)
A/wild chicken/Shanghai/C1/2014(H9N2)
A/chicken/Zhejiang/3C9/2014(H9N2)
A/chicken/Zhejiang/925122/2014(H9N2)
A/chicken/Shaoxing/5453/2013(H9N2)
A/chicken/Huzhou/3861/2013(H9N2)
A/pigeon/Zhejiang/77037/2014(H9N2)
A/chicken/Zhejiang/77024/2014(H6N2)
A/chicken/Zhejiang/77001/2014(H6N2)
A/chicken/Zhejiang/77025/2014(H6N2)
A/duck/Zhejiang/77174/2014(H6N2)
A/duck/Zhejiang/727038/2014(H6N2)
H9N2-like
0.02

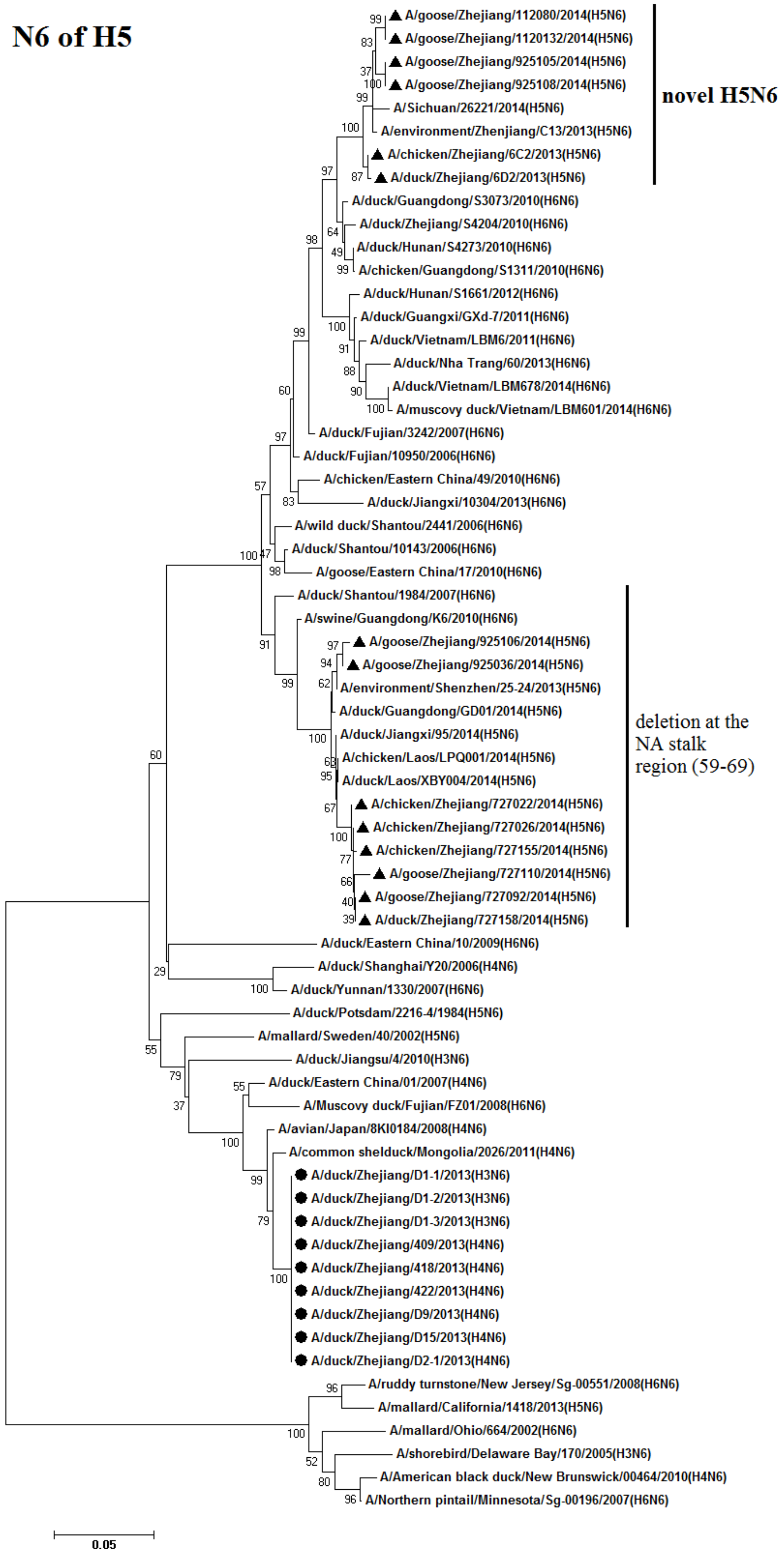
N6 of H5
A/goose/Zhejiang/112080/2014(H5N6)
A/goose/Zhejiang/1120132/2014(H5N6)
A/goose/Zhejiang/925105/2014(H5N6)
A/goose/Zhejiang/925108/2014(H5N6)
A/Sichuan/26221/2014(H5N6)
A/environment/Zhenjiang/C13/2013(H5N6)
A/chicken/Zhejiang/6C2/2013(H5N6)
A/duck/Zhejiang/6D2/2013(H5N6)
novel H5N6
A/duck/Guangdong/S3073/2010(H6N6)
A/duck/Zhejiang/S4204/2010(H6N6)
A/duck/Hunan/S4273/2010(H6N6)
A/chicken/Guangdong/S1311/2010(H6N6)
A/duck/Hunan/S1661/2012(H6N6)
A/duck/Guangxi/GXd-7/2011(H6N6)
A/duck/Vietnam/LBM6/2011(H6N6)
A/duck/Nha Trang/60/2013(H6N6)
A/duck/Vietnam/LBM678/2014(H6N6)
A/muscovy duck/Vietnam/LBM601/2014(H6N6)
A/duck/Fujian/3242/2007(H6N6)
A/duck/Fujian/10950/2006(H6N6)
A/chicken/Eastern China/49/2010(H6N6)
A/duck/Jiangxi/10304/2013(H6N6)
A/wild duck/Shantou/2441/2006(H6N6)
A/duck/Shantou/10143/2006(H6N6)
A/goose/Eastern China/17/2010(H6N6)
A/duck/Shantou/1984/2007(H6N6)
A/swine/Guangdong/K6/2010(H6N6)
A/goose/Zhejiang/925106/2014(H5N6)
A/goose/Zhejiang/925036/2014(H5N6)
A/environment/Shenzhen/25-24/2013(H5N6)
A/duck/Guangdong/GD01/2014(H5N6)
A/duck/Jiangxi/95/2014(H5N6)
A/chicken/Laos/LPQ001/2014(H5N6)
A/duck/Laos/XBY004/2014(H5N6)
A/chicken/Zhejiang/727022/2014(H5N6)
A/chicken/Zhejiang/727026/2014(H5N6)
A/chicken/Zhejiang/727155/2014(H5N6)
A/goose/Zhejiang/727110/2014(H5N6)
A/goose/Zhejiang/727092/2014(H5N6)
A/duck/Zhejiang/727158/2014(H5N6)
deletion at the
NA stalk
region (59-69)
A/duck/Eastern China/10/2009(H6N6)
A/duck/Shanghai/Y20/2006(H4N6)
A/duck/Yunnan/1330/2007(H6N6)
A/duck/Potsdam/2216-4/1984(H5N6)
A/mallard/Sweden/40/2002(H5N6)
A/duck/Jiangsu/4/2010(H3N6)
A/duck/Eastern China/01/2007(H4N6)
A/Muscovy duck/Fujian/FZ01/2008(H6N6)
A/avian/Japan/8KI0184/2008(H4N6)
A/common shelduck/Mongolia/2026/2011(H4N6)
A/duck/Zhejiang/D1-1/2013(H3N6)
A/duck/Zhejiang/D1-2/2013(H3N6)
A/duck/Zhejiang/D1-3/2013(H3N6)
A/duck/Zhejiang/409/2013(H4N6)
A/duck/Zhejiang/418/2013(H4N6)
A/duck/Zhejiang/422/2013(H4N6)
A/duck/Zhejiang/D9/2013(H4N6)
A/duck/Zhejiang/D15/2013(H4N6)
A/duck/Zhejiang/D2-1/2013(H4N6)
A/ruddy turnstone/New Jersey/Sg-00551/2008(H6N6)
A/mallard/California/1418/2013(H5N6)
A/mallard/Ohio/664/2002(H6N6)
A/shorebird/Delaware Bay/170/2005(H3N6)
A/American black duck/New Brunswick/00464/2010(H4N6)
A/Northern pintail/Minnesota/Sg-00196/2007(H6N6)
0.05

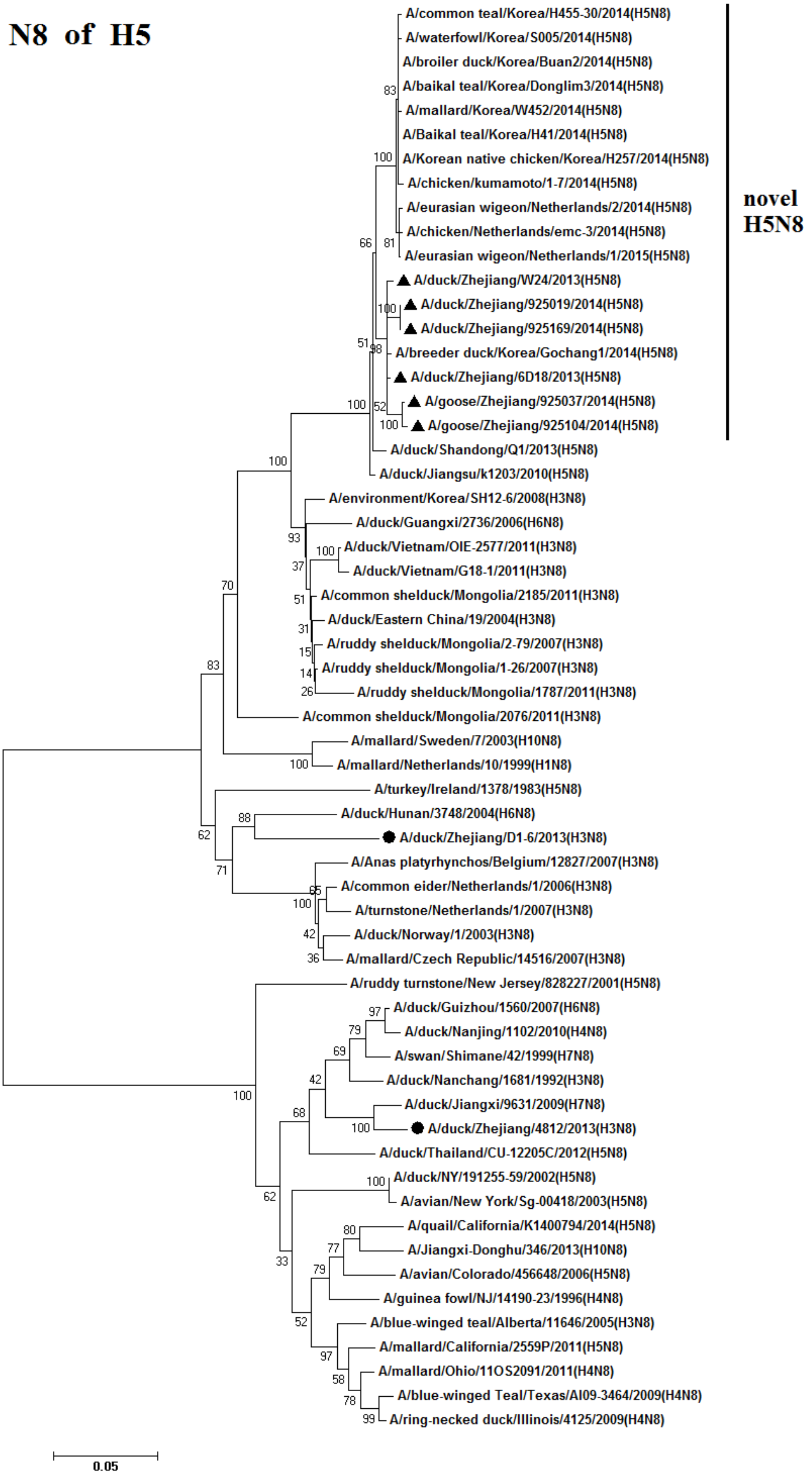
N8 of H5
A/common teal/Korea/H455-30/2014(H5N8)
A/waterfowl/Korea/S005/2014(H5N8)
A/broiler duck/Korea/Buan2/2014(H5N8)
A/baikal teal/Korea/Donglim3/2014(H5N8)
A/mallard/Korea/W452/2014(H5N8)
A/Baikal teal/Korea/H41/2014(H5N8)
A/Korean native chicken/Korea/H257/2014(H5N8)
A/chicken/kumamoto/1-7/2014(H5N8)
A/eurasian wigeon/Netherlands/2/2014(H5N8)
A/chicken/Netherlands/emc-3/2014(H5N8)
A/eurasian wigeon/Netherlands/1/2015(H5N8)
A/duck/Zhejiang/W24/2013(H5N8)
A/duck/Zhejiang/925019/2014(H5N8)
A/duck/Zhejiang/925169/2014(H5N8)
A/breeder duck/Korea/Gochang1/2014(H5N8)
A/duck/Zhejiang/6D18/2013(H5N8)
A/goose/Zhejiang/925037/2014(H5N8)
A/goose/Zhejiang/925104/2014(H5N8)
A/duck/Shandong/Q1/2013(H5N8)
A/duck/Jiangsu/k1203/2010(H5N8)
novel H5N8
A/environment/Korea/SH12-6/2008(H3N8)
A/duck/Guangxi/2736/2006(H6N8)
A/duck/Vietnam/OIE-2577/2011(H3N8)
A/duck/Vietnam/G18-1/2011(H3N8)
A/common shelduck/Mongolia/2185/2011(H3N8)
A/duck/Eastern China/19/2004(H3N8)
A/ruddy shelduck/Mongolia/2-79/2007(H3N8)
A/ruddy shelduck/Mongolia/1-26/2007(H3N8)
A/ruddy shelduck/Mongolia/1787/2011(H3N8)
A/common shelduck/Mongolia/2076/2011(H3N8)
A/mallard/Sweden/7/2003(H10N8)
A/mallard/Netherlands/10/1999(H1N8)
A/turkey/Ireland/1378/1983(H5N8)
A/duck/Hunan/3748/2004(H6N8)
A/duck/Zhejiang/D1-6/2013(H3N8)
A/Anas platyrhynchos/Belgium/12827/2007(H3N8)
A/common eider/Netherlands/1/2006(H3N8)
A/turnstone/Netherlands/1/2007(H3N8)
A/duck/Norway/1/2003(H3N8)
A/mallard/Czech Republic/14516/2007(H3N8)
A/ruddy turnstone/New Jersey/828227/2001(H5N8)
A/duck/Guizhou/1560/2007(H6N8)
A/duck/Nanjing/1102/2010(H4N8)
A/swan/Shimane/42/1999(H7N8)
A/duck/Nanchang/1681/1992(H3N8)
A/duck/Jiangxi/9631/2009(H7N8)
A/duck/Zhejiang/4812/2013(H3N8)
A/duck/Thailand/CU-12205C/2012(H5N8)
A/duck/NY/191255-59/2002(H5N8)
A/avian/New York/Sg-00418/2003(H5N8)
A/quail/California/K1400794/2014(H5N8)
A/Jiangxi-Donghu/346/2013(H10N8)
A/avian/Colorado/456648/2006(H5N8)
A/guinea fowl/NJ/14190-23/1996(H4N8)
A/blue-winged teal/Alberta/11646/2005(H3N8)
A/mallard/California/2559P/2011(H5N8)
A/mallard/Ohio/11OS2091/2011(H4N8)
A/blue-winged Teal/Texas/AI09-3464/2009(H4N8)
A/ring-necked duck/Illinois/4125/2009(H4N8)
0.05

PB2 of H5

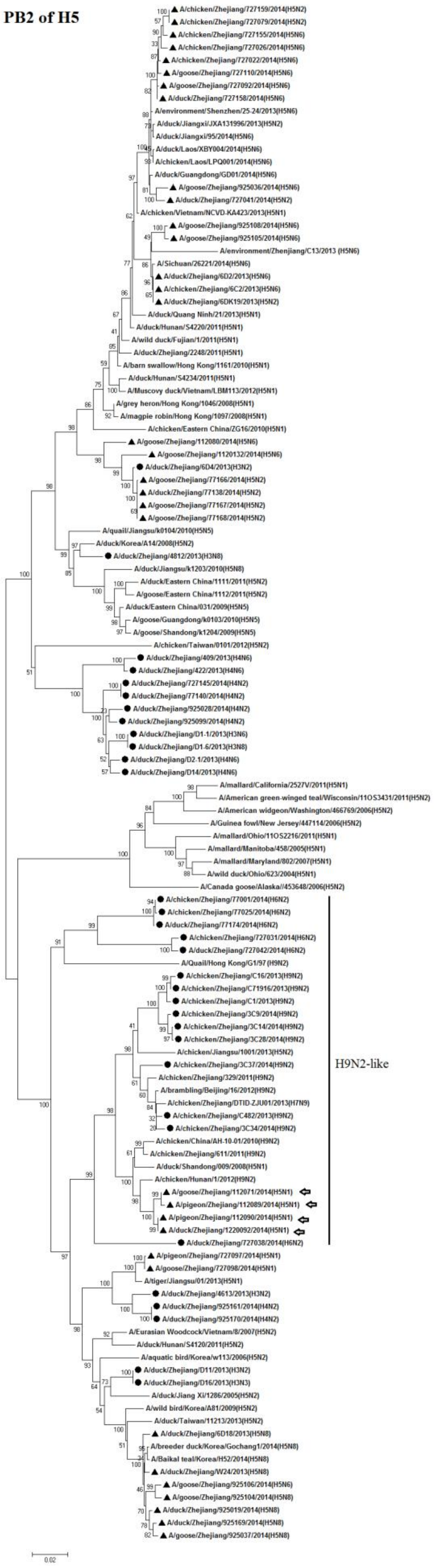

A/chicken/Zhejiang/727159/2014(H5N2)
A/chicken/Zhejiang/727079/2014(H5N2)
A/chicken/Zhejiang/727155/2014(H5N6)
A/chicken/Zhejiang/727026/2014(H5N6)
A/chicken/Zhejiang/727022/2014(H5N6)
A/goose/Zhejiang/727110/2014(H5N6)
A/goose/Zhejiang/727092/2014(H5N6)
A/duck/Zhejiang/727158/2014(H5N6)
A/environment/Shenzhen/25-24/2013(H5N6)
A/duck/Jiangxi/JXA131996/2013(H5N2)
A/duck/Jiangxi/95/2014(H5N6)
A/duck/Laos/XBY004/2014(H5N6)
A/chicken/Laos/LPQ001/2014(H5N6)
A/duck/Guangdong/GD01/2014(H5N6)
A/goose/Zhejiang/925036/2014(H5N6)
A/duck/Zhejiang/727041/2014(H5N2)
A/chicken/Vietnam/NCVD-KA423/2013(H5N1)
A/goose/Zhejiang/925108/2014(H5N6)
A/goose/Zhejiang/925105/2014(H5N6)
A/environment/Zhenjiang/C13/2013 (H5N6)
A/Sichuan/26221/2014(H5N6)
A/duck/Zhejiang/6D2/2013(H5N6)
A/chicken/Zhejiang/6C2/2013(H5N6)
A/duck/Zhejiang/6DK19/2013(H5N2)
A/duck/Quang Ninh/21/2013(H5N1)
A/duck/Hunan/S4220/2011(H5N1)
A/wild duck/Fujian/1/2011(H5N1)
A/duck/Zhejiang/2248/2011(H5N1)
A/barn swallow/Hong Kong/1161/2010(H5N1)
A/duck/Hunan/S4234/2011(H5N1)
A/Muscovy duck/Vietnam/LBM113/2012(H5N1)
A/grey heron/Hong Kong/1046/2008(H5N1)
A/magpie robin/Hong Kong/1097/2008(H5N1)
A/chicken/Eastern China/ZG16/2010(H5N1)
A/goose/Zhejiang/112080/2014(H5N6)
A/goose/Zhejiang/1120132/2014(H5N6)
A/duck/Zhejiang/6D4/2013(H3N2)
A/goose/Zhejiang/77166/2014(H5N2)
A/duck/Zhejiang/77138/2014(H5N2)
A/goose/Zhejiang/77167/2014(H5N2)
A/goose/Zhejiang/77168/2014(H5N2)
A/quail/Jiangsu/k0104/2010(H5N5)
A/duck/Korea/A14/2008(H5N2)
A/duck/Zhejiang/4812/2013(H3N8)
A/duck/Jiangsu/k1203/2010(H5N8)
A/duck/Eastern China/1111/2011(H5N2)
A/goose/Eastern China/1112/2011(H5N2)
A/duck/Eastern China/031/2009(H5N5)
A/goose/Guangdong/k0103/2010(H5N5)
A/goose/Shandong/k1204/2009(H5N5)
A/chicken/Taiwan/0101/2012(H5N2)
A/duck/Zhejiang/409/2013(H4N6)
A/duck/Zhejiang/422/2013(H4N6)
A/duck/Zhejiang/727145/2014(H4N2)
A/duck/Zhejiang/77140/2014(H4N2)
A/duck/Zhejiang/925028/2014(H4N2)
A/duck/Zhejiang/925099/2014(H4N2)
A/duck/Zhejiang/D1-1/2013(H3N6)
A/duck/Zhejiang/D1-6/2013(H3N8)
A/duck/Zhejiang/D2-1/2013(H4N6)
A/duck/Zhejiang/D14/2013(H4N6)
A/mallard/California/2527V/2011(H5N1)
A/American green-winged teal/Wisconsin/11OS3431/2011(H5N2)
A/American widgeon/Washington/466769/2006(H5N2)
A/Guinea fowl/New Jersey/447114/2006(H5N2)
A/mallard/Ohio/11OS2216/2011(H5N1)
A/mallard/Manitoba/458/2005(H5N1)
A/mallard/Maryland/802/2007(H5N1)
A/wild duck/Ohio/623/2004(H5N1)
A/Canada goose/Alaska//453648/2006(H5N2)
A/chicken/Zhejiang/77001/2014(H6N2)
A/chicken/Zhejiang/77025/2014(H6N2)
A/duck/Zhejiang/77174/2014(H6N2)
A/chicken/Zhejiang/727031/2014(H6N2)
A/duck/Zhejiang/727042/2014(H6N2)
A/Quail/Hong Kong/G1/97 (H9N2)
A/chicken/Zhejiang/C16/2013(H9N2)
A/chicken/Zhejiang/C71916/2013(H9N2)
A/chicken/Zhejiang/C1/2013(H9N2)
A/chicken/Zhejiang/3C9/2014(H9N2)
A/chicken/Zhejiang/3C14/2014(H9N2)
A/chicken/Zhejiang/3C28/2014(H9N2)
A/chicken/Jiangsu/1001/2013(H5N2)
A/chicken/Zhejiang/3C37/2014(H9N2)
A/chicken/Zhejiang/329/2011(H9N2)
A/brambling/Beijing/16/2012(H9N2)
A/chicken/Zhejiang/DTID-ZJU01/2013(H7N9)
A/chicken/Zhejiang/C482/2013(H9N2)
A/chicken/Zhejiang/3C34/2014(H9N2)
A/chicken/China/AH-10-01/2010(H9N2)
A/chicken/Zhejiang/611/2011(H9N2)
A/duck/Shandong/009/2008(H5N1)
A/chicken/Hunan/1/2012(H9N2)
A/goose/Zhejiang/112071/2014(H5N1)
A/pigeon/Zhejiang/112089/2014(H5N1)
A/pigeon/Zhejiang/112090/2014(H5N1)
A/duck/Zhejiang/1220092/2014(H5N1)
A/duck/Zhejiang/727038/2014(H6N2)
H9N2-like
A/pigeon/Zhejiang/727097/2014(H5N1)
A/goose/Zhejiang/727098/2014(H5N1)
A/tiger/Jiangsu/01/2013(H5N1)
A/duck/Zhejiang/4613/2013(H3N2)
A/duck/Zhejiang/925161/2014(H4N2)
A/duck/Zhejiang/925170/2014(H4N2)
A/Eurasian Woodcock/Vietnam/8/2007(H5N2)
A/duck/Hunan/S4120/2011(H5N2)
A/aquatic bird/Korea/w113/2006(H5N2)
A/duck/Zhejiang/D11/2013(H3N2)
A/duck/Zhejiang/D16/2013(H3N3)
A/duck/Jiang Xi/1286/2005(H5N2)
A/wild bird/Korea/A81/2009(H5N2)
A/duck/Taiwan/11213/2013(H5N2)
A/duck/Zhejiang/6D18/2013(H5N8)
A/breeder duck/Korea/Gochang1/2014(H5N8)
A/Baikal teal/Korea/H52/2014(H5N8)
A/duck/Zhejiang/W24/2013(H5N8)
A/goose/Zhejiang/925106/2014(H5N6)
A/goose/Zhejiang/925104/2014(H5N8)
A/duck/Zhejiang/925019/2014(H5N8)
A/duck/Zhejiang/925169/2014(H5N8)
A/goose/Zhejiang/925037/2014(H5N8)
0.02

PB1 of H5

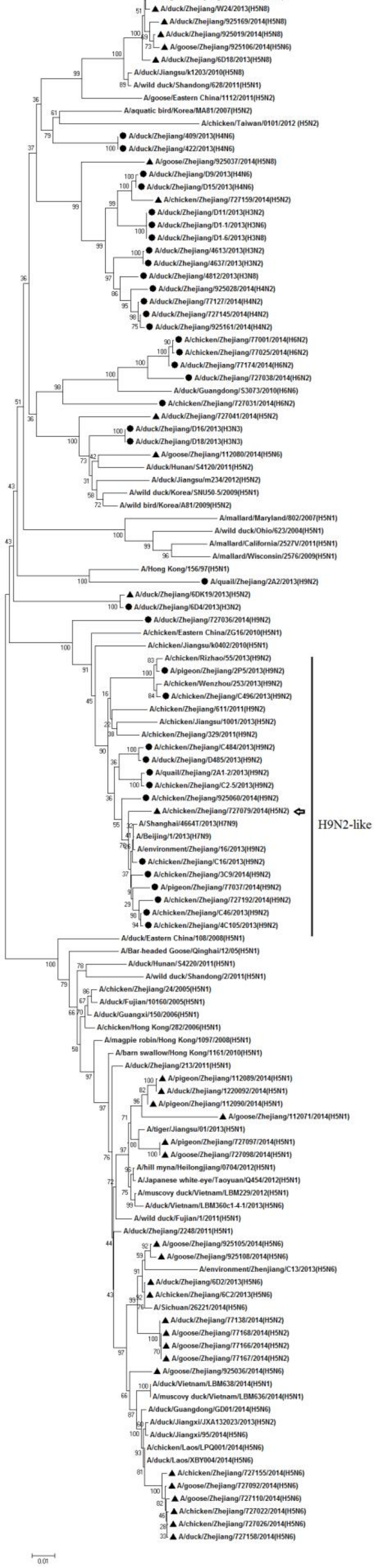
A/goose/Zhejiang/1120132/2014(H5N6)
A/goose/Zhejiang/925104/2014(H5N8)
A/duck/Zhejiang/W24/2013(H5N8)
A/duck/Zhejiang/925169/2014(H5N8)
A/duck/Zhejiang/925019/2014(H5N8)
A/goose/Zhejiang/925106/2014(H5N6)
A/duck/Zhejiang/6D18/2013(H5N8)
A/duck/Jiangsu/k1203/2010(H5N8)
A/wild duck/Shandong/628/2011(H5N1)
A/goose/Eastern China/1112/2011(H5N2)
A/aquatic bird/Korea/MA81/2007(H5N2)
A/chicken/Taiwan/0101/2012 (H5N2)
A/duck/Zhejiang/409/2013(H4N6)
A/duck/Zhejiang/422/2013(H4N6)
A/goose/Zhejiang/925037/2014(H5N8)
A/duck/Zhejiang/D9/2013(H4N6)
A/duck/Zhejiang/D15/2013(H4N6)
A/chicken/Zhejiang/727159/2014(H5N2)
A/duck/Zhejiang/D11/2013(H3N2)
A/duck/Zhejiang/D1-1/2013(H3N6)
A/duck/Zhejiang/D1-6/2013(H3N8)
A/duck/Zhejiang/4613/2013(H3N2)
A/duck/Zhejiang/4637/2013(H3N2)
A/duck/Zhejiang/4812/2013(H3N8)
A/duck/Zhejiang/925028/2014(H4N2)
A/duck/Zhejiang/77127/2014(H4N2)
A/duck/Zhejiang/727145/2014(H4N2)
A/duck/Zhejiang/925161/2014(H4N2)
A/chicken/Zhejiang/77001/2014(H6N2)
A/chicken/Zhejiang/77025/2014(H6N2)
A/duck/Zhejiang/77174/2014(H6N2)
A/duck/Zhejiang/727038/2014(H6N2)
A/duck/Guangdong/S3073/2010(H6N6)
A/chicken/Zhejiang/727031/2014(H6N2)
A/duck/Zhejiang/727041/2014(H5N2)
A/duck/Zhejiang/D16/2013(H3N3)
A/duck/Zhejiang/D18/2013(H3N3)
A/goose/Zhejiang/112080/2014(H5N6)
A/duck/Hunan/S4120/2011(H5N2)
A/duck/Jiangsu/m234/2012(H5N2)
A/wild duck/Korea/SNU50-5/2009(H5N1)
A/wild bird/Korea/A81/2009(H5N2)
A/mallard/Maryland/802/2007(H5N1)
A/wild duck/Ohio/623/2004(H5N1)
A/mallard/California/2527V/2011(H5N1)
A/mallard/Wisconsin/2576/2009(H5N1)
A/Hong Kong/156/97(H5N1)
A/quail/Zhejiang/2A2/2013(H9N2)
A/duck/Zhejiang/6DK19/2013(H5N2)
A/duck/Zhejiang/6D4/2013(H3N2)
A/duck/Zhejiang/727036/2014(H9N2)
A/chicken/Eastern China/ZG16/2010(H5N1)
A/chicken/Jiangsu/k0402/2010(H5N1)
A/chicken/Rizhao/55/2013(H9N2)
A/pigeon/Zhejiang/2P5/2013(H9N2)
A/chicken/Wenzhou/253/2013(H9N2)
A/chicken/Zhejiang/C496/2013(H9N2)
A/chicken/Zhejiang/611/2011(H9N2)
A/chicken/Jiangsu/1001/2013(H5N2)
A/chicken/Zhejiang/329/2011(H9N2)
A/chicken/Zhejiang/C484/2013(H9N2)
A/duck/Zhejiang/D485/2013(H9N2)
A/quail/Zhejiang/2A1-2/2013(H9N2)
A/chicken/Zhejiang/C2-5/2013(H9N2)
A/chicken/Zhejiang/925060/2014(H9N2)
A/chicken/Zhejiang/727079/2014(H5N2)
A/Shanghai/4664T/2013(H7N9)
A/Beijing/1/2013(H7N9)
A/environment/Zhejiang/16/2013(H9N2)
A/chicken/Zhejiang/C16/2013(H9N2)
A/chicken/Zhejiang/3C9/2014(H9N2)
A/pigeon/Zhejiang/77037/2014(H9N2)
A/chicken/Zhejiang/727192/2014(H9N2)
A/chicken/Zhejiang/C46/2013(H9N2)
A/chicken/Zhejiang/4C105/2013(H9N2)
H9N2-like
A/duck/Eastern China/108/2008(H5N1)
A/Bar-headed Goose/Qinghai/12/05(H5N1)
A/duck/Hunan/S4220/2011(H5N1)
A/wild duck/Shandong/2/2011(H5N1)
A/chicken/Zhejiang/24/2005(H5N1)
A/duck/Fujian/10160/2005(H5N1)
A/duck/Guangxi/150/2006(H5N1)
A/chicken/Hong Kong/282/2006(H5N1)
A/magpie robin/Hong Kong/1097/2008(H5N1)
A/barn swallow/Hong Kong/1161/2010(H5N1)
A/duck/Zhejiang/213/2011(H5N1)
A/pigeon/Zhejiang/112089/2014(H5N1)
A/duck/Zhejiang/1220092/2014(H5N1)
A/pigeon/Zhejiang/112090/2014(H5N1)
A/goose/Zhejiang/112071/2014(H5N1)
A/tiger/Jiangsu/01/2013(H5N1)
A/pigeon/Zhejiang/727097/2014(H5N1)
A/goose/Zhejiang/727098/2014(H5N1)
A/hill myna/Heilongjiang/0704/2012(H5N1)
A/Japanese white-eye/Taoyuan/Q454/2012(H5N1)
A/muscovy duck/Vietnam/LBM229/2012(H5N1)
A/duck/Vietnam/LBM360c1-4-1/2013(H5N6)
A/wild duck/Fujian/1/2011(H5N1)
A/duck/Zhejiang/2248/2011(H5N1)
A/goose/Zhejiang/925105/2014(H5N6)
A/goose/Zhejiang/925108/2014(H5N6)
A/environment/Zhenjiang/C13/2013(H5N6)
A/duck/Zhejiang/6D2/2013(H5N6)
A/chicken/Zhejiang/6C2/2013(H5N6)
A/Sichuan/26221/2014(H5N6)
A/duck/Zhejiang/77138/2014(H5N2)
A/goose/Zhejiang/77168/2014(H5N2)
A/goose/Zhejiang/77166/2014(H5N2)
A/goose/Zhejiang/77167/2014(H5N2)
A/goose/Zhejiang/925036/2014(H5N6)
A/duck/Vietnam/LBM638/2014(H5N1)
A/muscovy duck/Vietnam/LBM636/2014(H5N1)
A/duck/Guangdong/GD01/2014(H5N6)
A/duck/Jiangxi/JXA132023/2013(H5N2)
A/duck/Jiangxi/95/2014(H5N6)
A/chicken/Laos/LPQ001/2014(H5N6)
A/duck/Laos/XBY004/2014(H5N6)
A/chicken/Zhejiang/727155/2014(H5N6)
A/goose/Zhejiang/727092/2014(H5N6)
A/goose/Zhejiang/727110/2014(H5N6)
A/chicken/Zhejiang/727022/2014(H5N6)
A/chicken/Zhejiang/727026/2014(H5N6)
A/duck/Zhejiang/727158/2014(H5N6)
0.01

PA of H5

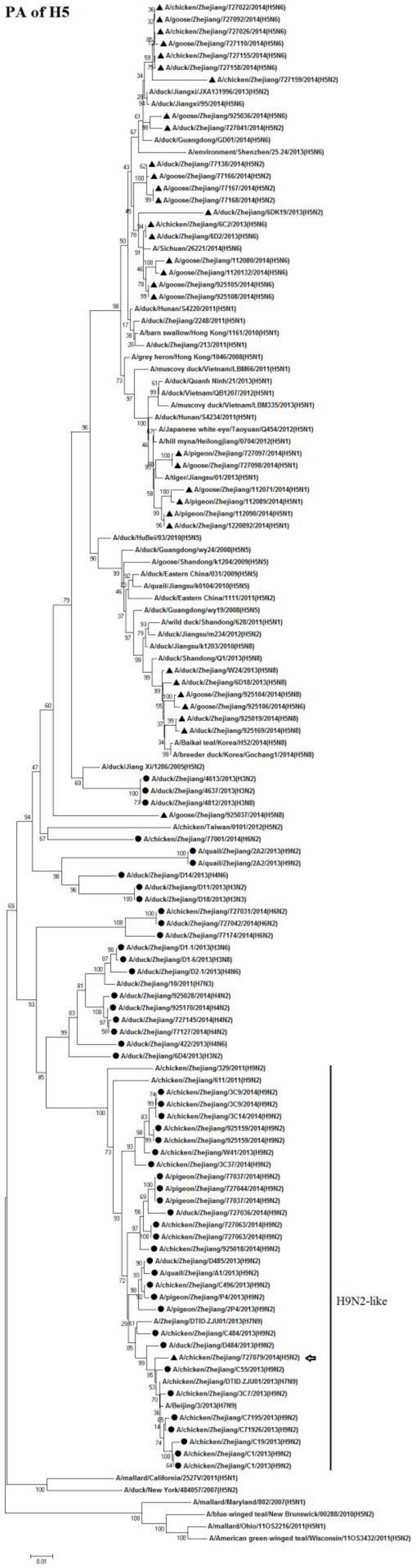

NP of H5

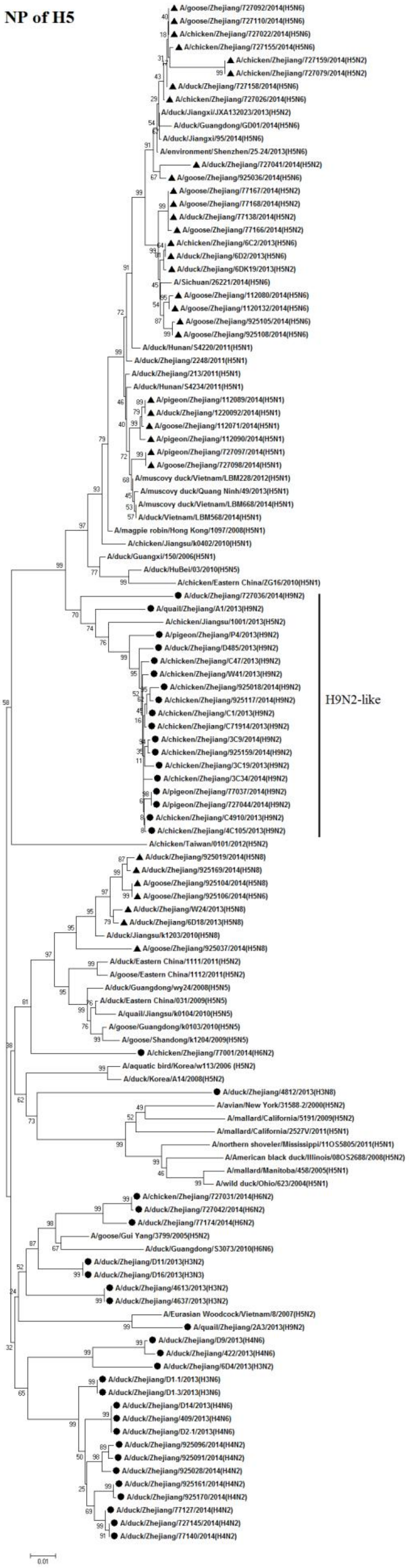

M of H5

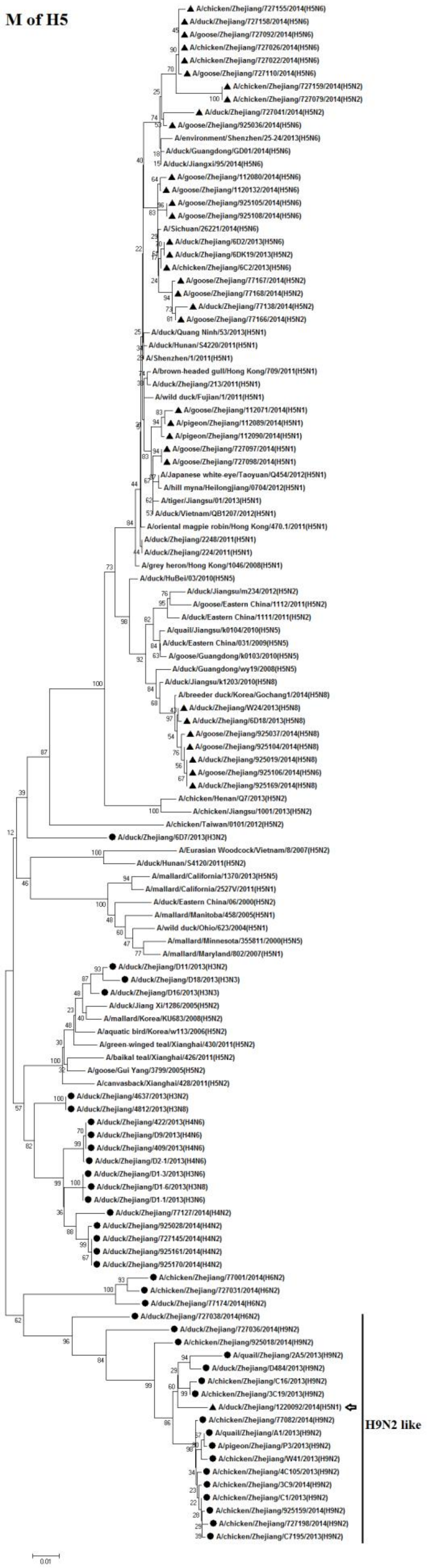

A/chicken/Zhejiang/727155/2014(H5N6)
A/duck/Zhejiang/727158/2014(H5N6)
A/goose/Zhejiang/727092/2014(H5N6)
A/chicken/Zhejiang/727026/2014(H5N6)
A/chicken/Zhejiang/727022/2014(H5N6)
A/goose/Zhejiang/727110/2014(H5N6)
A/chicken/Zhejiang/727159/2014(H5N2)
A/chicken/Zhejiang/727079/2014(H5N2)
A/duck/Zhejiang/727041/2014(H5N2)
A/goose/Zhejiang/925036/2014(H5N6)
A/environment/Shenzhen/25-24/2013(H5N6)
A/duck/Guangdong/GD01/2014(H5N6)
A/duck/Jiangxi/95/2014(H5N6)
A/goose/Zhejiang/112080/2014(H5N6)
A/goose/Zhejiang/1120132/2014(H5N6)
A/goose/Zhejiang/925105/2014(H5N6)
A/goose/Zhejiang/925108/2014(H5N6)
A/Sichuan/26221/2014(H5N6)
A/duck/Zhejiang/6D2/2013(H5N6)
A/duck/Zhejiang/6DK19/2013(H5N2)
A/chicken/Zhejiang/6C2/2013(H5N6)
A/goose/Zhejiang/77167/2014(H5N2)
A/goose/Zhejiang/77168/2014(H5N2)
A/duck/Zhejiang/77138/2014(H5N2)
A/goose/Zhejiang/77166/2014(H5N2)
A/duck/Quang Ninh/53/2013(H5N1)
A/duck/Hunan/S4220/2011(H5N1)
A/Shenzhen/1/2011(H5N1)
A/brown-headed gull/Hong Kong/709/2011(H5N1)
A/duck/Zhejiang/213/2011(H5N1)
A/wild duck/Fujian/1/2011(H5N1)
A/goose/Zhejiang/112071/2014(H5N1)
A/pigeon/Zhejiang/112089/2014(H5N1)
A/pigeon/Zhejiang/112090/2014(H5N1)
A/goose/Zhejiang/727097/2014(H5N1)
A/goose/Zhejiang/727098/2014(H5N1)
A/Japanese white-eye/Taoyuan/Q454/2012(H5N1)
A/hill myna/Heilongjiang/0704/2012(H5N1)
A/tiger/Jiangsu/01/2013(H5N1)
A/duck/Vietnam/QB1207/2012(H5N1)
A/oriental magpie robin/Hong Kong/470.1/2011(H5N1)
A/duck/Zhejiang/2248/2011(H5N1)
A/duck/Zhejiang/224/2011(H5N1)
A/grey heron/Hong Kong/1046/2008(H5N1)
A/duck/HuBei/03/2010(H5N5)
A/duck/Jiangsu/m234/2012(H5N2)
A/goose/Eastern China/1112/2011(H5N2)
A/duck/Eastern China/1111/2011(H5N2)
A/quail/Jiangsu/k0104/2010(H5N5)
A/duck/Eastern China/031/2009(H5N5)
A/goose/Guangdong/k0103/2010(H5N5)
A/duck/Guangdong/wy19/2008(H5N5)
A/duck/Jiangsu/k1203/2010(H5N8)
A/breeder duck/Korea/Gochang1/2014(H5N8)
A/duck/Zhejiang/W24/2013(H5N8)
A/duck/Zhejiang/6D18/2013(H5N8)
A/goose/Zhejiang/925037/2014(H5N8)
A/goose/Zhejiang/925104/2014(H5N8)
A/duck/Zhejiang/925019/2014(H5N8)
A/goose/Zhejiang/925106/2014(H5N6)
A/duck/Zhejiang/925169/2014(H5N8)
A/chicken/Henan/Q7/2013(H5N2)
A/chicken/Jiangsu/1001/2013(H5N2)
A/chicken/Taiwan/0101/2012(H5N2)
A/duck/Zhejiang/6D7/2013(H3N2)
A/Eurasian Woodcock/Vietnam/8/2007(H5N2)
A/duck/Hunan/S4120/2011(H5N2)
A/mallard/California/1370/2013(H5N5)
A/mallard/California/2527V/2011(H5N1)
A/duck/Eastern China/06/2000(H5N2)
A/mallard/Manitoba/458/2005(H5N1)
A/wild duck/Ohio/623/2004(H5N1)
A/mallard/Minnesota/355811/2000(H5N5)
A/mallard/Maryland/802/2007(H5N1)
A/duck/Zhejiang/D11/2013(H3N2)
A/duck/Zhejiang/D18/2013(H3N3)
A/duck/Zhejiang/D16/2013(H3N3)
A/duck/Jiang Xi/1286/2005(H5N2)
A/mallard/Korea/KU683/2008(H5N2)
A/aquatic bird/Korea/w113/2006(H5N2)
A/green-winged teal/Xianghai/430/2011(H5N2)
A/baikal teal/Xianghai/426/2011(H5N2)
A/goose/Gui Yang/3799/2005(H5N2)
A/canvasback/Xianghai/428/2011(H5N2)
A/duck/Zhejiang/4637/2013(H3N2)
A/duck/Zhejiang/4812/2013(H3N8)
A/duck/Zhejiang/422/2013(H4N6)
A/duck/Zhejiang/D9/2013(H4N6)
A/duck/Zhejiang/409/2013(H4N6)
A/duck/Zhejiang/D2-1/2013(H4N6)
A/duck/Zhejiang/D1-3/2013(H3N6)
A/duck/Zhejiang/D1-6/2013(H3N8)
A/duck/Zhejiang/D1-1/2013(H3N6)
A/duck/Zhejiang/77127/2014(H4N2)
A/duck/Zhejiang/925028/2014(H4N2)
A/duck/Zhejiang/727145/2014(H4N2)
A/duck/Zhejiang/925161/2014(H4N2)
A/duck/Zhejiang/925170/2014(H4N2)
A/chicken/Zhejiang/77001/2014(H6N2)
A/chicken/Zhejiang/727031/2014(H6N2)
A/duck/Zhejiang/77174/2014(H6N2)
A/duck/Zhejiang/727038/2014(H6N2)
A/duck/Zhejiang/727036/2014(H9N2)
A/chicken/Zhejiang/925018/2014(H9N2)
A/quail/Zhejiang/2A5/2013(H9N2)
A/duck/Zhejiang/D484/2013(H9N2)
A/chicken/Zhejiang/C16/2013(H9N2)
A/chicken/Zhejiang/3C19/2013(H9N2)
A/duck/Zhejiang/1220092/2014(H5N1)
A/chicken/Zhejiang/77082/2014(H9N2)
A/quail/Zhejiang/A1/2013(H9N2)
A/pigeon/Zhejiang/P3/2013(H9N2)
A/chicken/Zhejiang/W41/2013(H9N2)
A/chicken/Zhejiang/4C105/2013(H9N2)
A/chicken/Zhejiang/3C9/2014(H9N2)
A/chicken/Zhejiang/C1/2013(H9N2)
A/chicken/Zhejiang/925159/2014(H9N2)
A/chicken/Zhejiang/727198/2014(H9N2)
A/chicken/Zhejiang/C7195/2013(H9N2)
H9N2 like
0.01

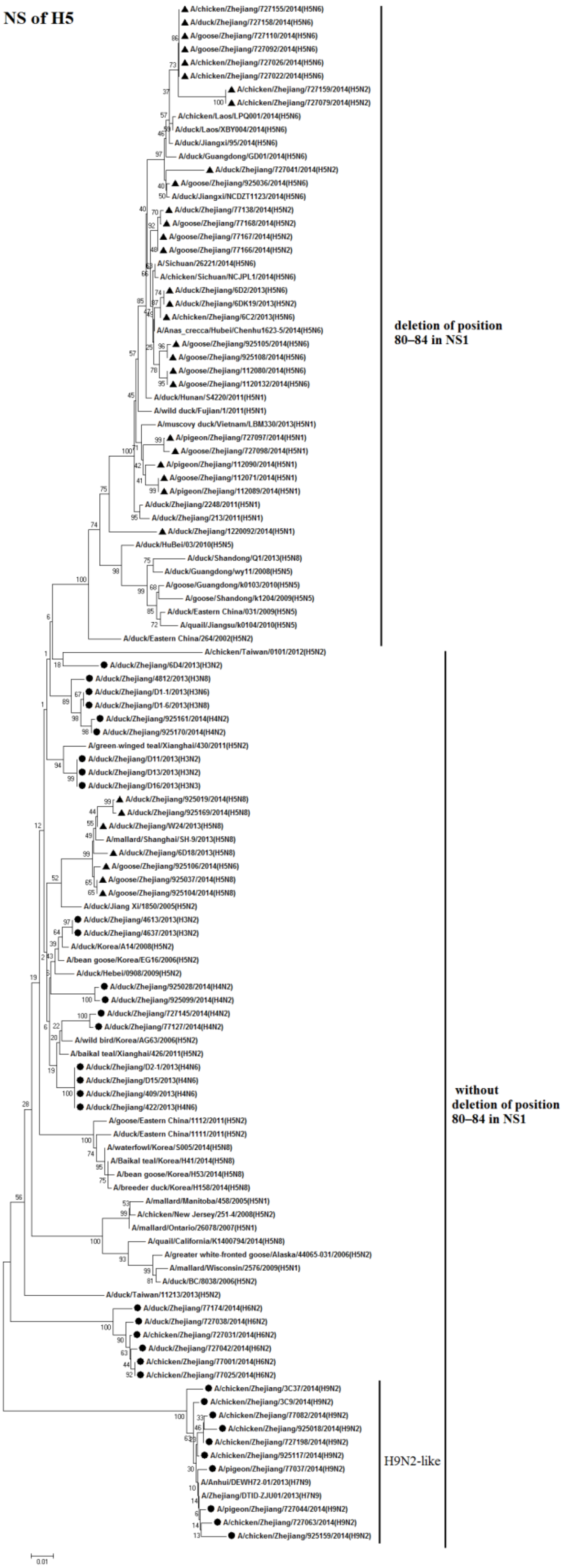

NS of H5
deletion of position
80–84 in NS1
without
deletion of position
80–84 in NS1
H9N2-like
0.01

Table S1. Genotype descriptions of the classical reference viruses and H9N2 avian influenza virus field isolates

**Representative isolates**[a]	**Lineage**[b] **at the following gene segment**								**Genotypes**
	**PB2**	**PB1**	**PA**	**HA**	**NP**	**NA**	**M**	**NS**	
CK/BJ/1/94	BJ94	BJ94	BJ94	BJ94	BJ94	BJ94	BJ94	BJ94	A
CK/GD/6/97	G1	G1	BJ94	BJ94	BJ94	G9	BJ94	BJ94	B
CK/GX/10/99	G1	G1	G1	BJ94	BJ94	G9	BJ94	G1	C
CK/GX/9/99	G1	G1	G1	BJ94	BJ94	BJ94	BJ94	G1	D
CK/HLJ35//00	G1	G1	G1	Ws66	Ws66	G9	BJ94	BJ94	E
CK/GD/10/00	BJ94	BJ94	BJ94	BJ94	BJ94	G9	BJ94	BJ94	F
CK/HN/26/00	G1	BJ94	BJ94	BJ94	BJ94	BJ94	BJ94	BJ94	G
CK/SH/F/98	SH98	SH98	SH98	BJ94	SH98	BJ94	BJ94	BJ94	H
CK/GD/56/01	SH98	SH98	SH98	BJ94	SH98	G9	BJ94	BJ94	I
CK/JS/1/99	SH98	SH98	SH98	BJ94	SH98	BJ94	BJ94	HK76	J
CK/SD/1/03	BJ94	BJ94	Kor96	BJ94	BJ94	BJ94	BJ94	BJ94	K
DK/Js/3/05	SH98	SH98	SH98	BJ94	SH98	BJ94	BJ94	Kor96	L
CK/SD/B1/98	BJ94	BJ94	SH98	BJ94	BJ94	BJ94	BJ94	BJ94	M
CK/SD/B2/07	BJ94	SH98	SH98	BJ94	SH98	BJ94	BJ94	BJ94	N
CK/SD/B4/07	SH98	SH98	SH98	BJ94	SH98	BJ94	G1	BJ94	O
CK/ZB/B1/08	SH98	SH98	SH98	BJ94	SH98	G9	G1	BJ94	P
CK/HN/L1/08	SH98	BJ94	Y439	BJ94	SH98	G9	G1	BJ94	Q
A/chicken/Zhejiang/C1/2013	G1	SH98	SH98	BJ94	SH98	BJ94	G1	BJ94	S

A/quail/Zhejiang/2A2/2013	G1	G1	Y439	BJ94	G1	G1	G1	G1	Novel

Note: Viruses were harvested during surveillance of live poultry markets in Zhejiang, China from January 2013 through December 2014.

[a] Genotypes of representative isolates were assigned as described previously [7, 26, 27, 49].

[b] The lineage for each viral gene segment is coded as: BJ94= Ck/BJ/1/94-like genes; SH98= Ck/SH/F/98-like genes; G1= Qa/HK/G1/97-like genes; Y439= Dk/HK/Y439/97-like genes; HK76= DK/HK/d73/76-like genes; Kor96= Ck/Kor/323/96-like genes.

Table S2. Comparison of amino acid sequences of HA and NA genes of H9N2 avian influenza viruses isolated from poultry in Zhejiang, China.

Representative isolates	Amino acids at the receptor-binding sites		Cleavage site	NA deletion
	Selected locations[a]	Left edge aa 232–237	aa 333–340	(aa)
A/chicken/Zhejiang/C1/2013	YWTN***A***LY	NGLMGR	PSRSSR/GL	62–64
A/chicken/Zhejiang/C19/2013	YWTN***A***LY	NGLMGR	PSRSSR/GL	62–64
A/chicken/Zhejiang/C71916/2013	YWTN***A***LY	NGLMGR	PSRSSR/GL	62–64
A/chicken/Zhejiang/C16/2013	YWTNTLY	NGLMGR	PS***K***SSR/GL	62–64
A/chicken/Zhejiang/727198/2014	YWTNTLY	NGLMGR	PSRSSR/GL	62–64
A/chicken/Zhejiang/925117/2014	YWTNTLY	NGLMGR	PSRSSR/GL	62–64
A/quail/Zhejiang/2A5/2013	YWTN***V***LY	NGLMGR	PSRSSR/GL	62–64
A/quail/Zhejiang/2A2/2013	YWTNTLY	NGLMGR	PSRSSR/GL	38–39
A/quail/Zhejiang/2A3/2013	YWTNTLY	NGLMGR	PSRSSR/GL	38–39
A/quail/Zhejiang/2A4/2013	YWTNTLY	NGLMGR	PSRSSR/GL	38–39
Ck/BJ/1/94	YWTN***V***LY	NG***QQ***GR	P***A***RSSR/GL	NO
Ck/SH/F/98	YWTN***A***LY	NG***QQ***GR	P***A***RSSR/GL	62–64
Qa/HK/ G1/97	YWT***HE***LY	N***D***L***Q***GR	P***A***RSSR/GL	38–39
Dk/HK/Y439/97	YWT***HE***LY	N***DQQ***GR	P***AA***S***N***R/GL	NO
Dk/HK/Y280/97	YWTNTLY	NGL***Q***GR	P***A***RSSR/GL	62–64
A/turkey/Wisconsin/1/1966	YWT***HE***LY	NG***QQ***GR	P***AV***SSR/GL	NO

Note: [a] From left to right, [aa] 109, 161, 163, 191, 198, 202, and 203.

Residues in ***bold italics*** indicate changes from the consensus alignment.

Table S3. Novel H5 avian influenza virus strains isolated from poultry.

No.	Strain	Subtype	HA clade	District, city	Date of sample collection	Species	H5 virus isolation rate (%)
1	A/pigeon/Zhejiang/727097/2014(H5N1)	H5N1	2.3.2	Yuhang, Hangzhou	Jul 27, 2014	pigeon	1.17% (3/255)*
2	A/pigeon/Zhejiang/112089/2014(H5N1)	H5N1	2.3.2	Haining, Jiaxing	Nov 20, 2014		
3	A/pigeon/Zhejiang/112090/2014(H5N1)	H5N1	2.3.2	Haining, Jiaxing	Nov 20, 2014		
4	A/chicken/Zhejiang/727079/2014(H5N2)	H5N2	2.3.4.6	Yuhang, Hangzhou	Jul 27, 2014	chicken	0.29% (6/2031)
5	A/chicken/Zhejiang/727159/2014(H5N2)	H5N2	2.3.4.6	Yuhang, Hangzhou	Jul 27, 2014		
6	A/chicken/Zhejiang/6C2/2013(H5N6)	H5N6	2.3.4.6	Anji, Huzhou	Dec 14, 2013		
7	A/chicken/Zhejiang/727022/2014(H5N6)	H5N6	2.3.4.6	Yuhang, Hangzhou	Jul 27, 2014		
8	A/chicken/Zhejiang/727026/2014(H5N6)	H5N6	2.3.4.6	Yuhang, Hangzhou	Jul 27, 2014		
9	A/chicken/Zhejiang/727155/2014(H5N6)	H5N6	2.3.4.6	Yuhang, Hangzhou	Jul 27, 2014		
10	A/duck/Zhejiang/1220092/2014(H5N1)	H5N1	2.3.2	Tongxiang, Jiaxing	Dec 25, 2014	duck	1.07% (10/929)
11	A/duck/Zhejiang/6DK19/2013(H5N2)	H5N2	2.3.4.6	Yuhang, Hangzhou	Dec 14, 2013		
12	A/duck/Zhejiang/77138/2014(H5N2)	H5N2	2.3.4.6	Tongxiang, Jiaxing	Jul 7, 2014		
13	A/duck/Zhejiang/727041/2014(H5N2)	H5N2	2.3.4.6	Yuhang, Hangzhou	Jul 27, 2014		
14	A/duck/Zhejiang/6D2/2013(H5N6)	H5N6	2.3.4.6	Anji, Huzhou	Dec 14, 2013		
15	A/duck/Zhejiang/727158/2014(H5N6)	H5N6	2.3.4.6	Yuhang, Hangzhou	Jul 27, 2014		
16	A/duck/Zhejiang/W24/2013(H5N8)	H5N8	2.3.4.6	Tongxiang, Jiaxing	Nov 14, 2013		
17	A/duck/Zhejiang/6D18/2013(H5N8)	H5N8	2.3.4.6	Yuhang, Hangzhou	Dec 14, 2013		
18	A/duck/Zhejiang/925169/2014(H5N8)	H5N8	2.3.4.6	Deqing, Huzhou	Sep 25, 2014		
19	A/duck/Zhejiang/925019/2014(H5N8)	H5N8	2.3.4.6	Deqing, Huzhou	Sep 25, 2014		

20	A/goose/Zhejiang/727098/2014(H5N1)	H5N1	2.3.2	Yuhang, Hangzhou	Jul 27,2014	goose	30% (15/50)
21	A/goose/Zhejiang/112071/2014(H5N1)	H5N1	2.3.2	Haining, Jiaxing	Nov 20,2014		
22	A/goose/Zhejiang/77166/2014(H5N2)	H5N2	2.3.4.6	Tongxiang, Jiaxing	Jul 7, 2014		
23	A/goose/Zhejiang/77167/2014(H5N2)	H5N2	2.3.4.6	Tongxiang, Jiaxing	Jul 7, 2014		
24	A/goose/Zhejiang/77168/2014(H5N2)	H5N2	2.3.4.6	Tongxiang, Jiaxing	Jul 7, 2014		
25	A/goose/Zhejiang/727092/2014(H5N6)	H5N6	2.3.4.6	Yuhang, Hangzhou	Jul 27, 2014		
26	A/goose/Zhejiang/727110/2014(H5N6)	H5N6	2.3.4.6	Yuhang, Hangzhou	Jul 27, 2014		
27	A/goose/Zhejiang/925105/2014(H5N6)	H5N6	2.3.4.6	Yuhang, Hangzhou	Sep 25, 2014		
28	A/goose/Zhejiang/925108/2014(H5N6)	H5N6	2.3.4.6	Yuhang, Hangzhou	Sep 25, 2014		
29	A/goose/Zhejiang/925106/2014(H5N6)	H5N6	2.3.4.6	Yuhang, Hangzhou	Sep 25, 2014		
30	A/goose/Zhejiang/925036/2014(H5N6)	H5N6	2.3.4.6	Yuhang, Hangzhou	Sep 25, 2014		
31	A/goose/Zhejiang/112080/2014(H5N6)	H5N6	2.3.4.6	Haining, Jiaxing	Nov 20, 2014		
32	A/goose/Zhejiang/1120132/2014(H5N6)	H5N6	2.3.4.6	Haining, Jiaxing	Nov 20, 2014		
33	A/goose/Zhejiang/925037/2014(H5N8)	H5N8	2.3.4.6	Yuhang, Hangzhou	Sep 25, 2014		
34	A/goose/Zhejiang/925104/2014(H5N8)	H5N8	2.3.4.6	Yuhang, Hangzhou	Sep 25, 2014		

Note: Viruses were harvested during surveillance of live poultry markets in Zhejiang, China from January 2013 through December 2014
